# Structures, stabilities and spectral properties of borospherene B_44_^−^ and metalloborospherenes MB_44_^0/−^ (M = Li, Na, and K)

**DOI:** 10.1038/srep40081

**Published:** 2017-01-10

**Authors:** Shixiong Li, Zhengping Zhang, Zhengwen Long, Shuijie Qin

**Affiliations:** 1 College of Big Data and Information Engineering, Guizhou University, Guiyang 550025, China; 2 School of Physics and Electronic Science, Guizhou Education University, Guiyang 550018, China; 3 College of Science, Guizhou University, Guiyang 550025, China; 4 Key Lab of Photoelectron Technology and Application, Guizhou University, Guiyang 550025, China

## Abstract

Density functional theory (DFT) and time-dependent density functional theory (TD-DFT) calculations are carried out to study the stabilities, photoelectron, infrared, Raman and electronic absorption spectra of borospherene B_44_^−^ and metalloborospherenes MB_44_^0/−^ (M = Li, Na, and K). It is found that all atoms can form stable exohedral metalloborospherenes M&B_44_^0/−^, whereas only Na and K atoms can be stably encapsulated inside B_44_^0/−^ cage. In addition, relative energies of these metalloborospherenes suggest that Na and K atoms favor exohedral configuration. Importantly, doping of metal atom can modify the stabilities of B_44_ with different structures, which provides a possible route to produce stable boron clusters or metalloborospherenes. The calculated results suggest that B_44_ tends to get electrons from the doped metal. Metalloborospherenes MB_44_^−^ are characterized as charge-transfer complexes (M^2+^B_44_^2−^), where B_44_ tends to get two electrons from the extra electron and the doped metal, resulting in similar features with anionic B_44_^2−^. In addition, doping of metal atom can change the spectral features, such as blueshift or redshift and weakening or strengthening of characteristic peaks, since the extra metal atom can modify the electronic structure. The calculated spectra are readily compared with future spectroscopy measurements and can be used as fingerprints to identify B_44_^−^ and metalloborospherenes.

Discovery of fullerene C_60_ is an important milestone in chemistry and materials science and leads to nowadays popular carbon-based nanomaterials such as carbon nanotubes and graphenes^1^,^2^,^3^,^4^. Fullerene C_60_ is spherical carbon cluster with a hollow cavity, which can encapsulate ion, atom or molecule inside the cage such as M@C_60_ (M = Li, N, H_2_, N_2_, and H_2_O) and M^+^@C_60_ (M = Li, H, Na, and K)^5^,^6^,^7^,^8^,^9^,^10^,^11^. These fullerene derivatives are known as endofullerenes which can produce new properties or improve the existing properties of fullerene C_60_. These endofullerenes based on C_60_ have attracted much attention due to their potential applications in materials science. Although boron is neighbor of carbon, boron is an electron deficient atom with only three valence electrons. Experimental and theoretical works have shown that most boron clusters are planar or quasi-planar structures^12^,^13^,^14^,^15^,^16^,^17^,^18^,^19^ before the intriguing fullerene-like cluster B_80_ predicted in 2007^20^. Subsequently, fullerene-like B_80_ was found not to be the global minimum, and the most favorable B_80_ is likely a core-shell type three-dimensional structure^21^,^22^. Since the first proposal of a possible B_80_ cage, the pursuit of all-boron fullerenes has attracted significant computational activities in the past several years^23^,^24^,^25^,^26^,^27^,^28^. Nevertheless, there has been no experimental evidence of the existence of all-boron fullerene in the past several decades.

An all-boron fullerene-like cluster B_40_^−^ and quasi-planar cluster B_40_^−^ were produced in a laser vaporization supersonic source in 2014^29^. Relevant theoretical simulations indicated that cage B_40_^−^ is slightly less stable than the quasi-planar global minimum B_40_^−^, however, neutral cage cluster B_40_ is the most stable structure among the isomers of B_40_. Photoelectron spectroscopy analysis indicated that combination of the simulated photoelectron spectra of cage B_40_^−^ and quasi-planar B_40_^−^ can reproduce the observed spectrum, which confirmed the existence of cage B_40_^−^. Soon after, the cage cluster B_39_^−^ was also produced via laser vaporization^30^. The first observation of the borospherene has aroused interest in all-boron fullerenes and their derivatives such as dynamical behavior of B_40_^31^, hydrogen storage capacity of Ti-decorated B_40_^32^, experimental and theoretical studies of B_28_^−^ and B_29_^−^ borospherenes^33^,^34^, structures and electronic properties of metalloborospherenes (Ca@B_40_, Be&B_40_, Sc@B_40_, Li&B_40_, Na@B_40_, Ca@B_39_^+^, Ca@B_38_, Ca@B_37_^−^ and Li_4_&B_36_)^35^,^36^,^37^,^38^,^39^,^40^,^41^, and spectral properties of borospherenes^42^,^43^.

Recently, a new borospherene B_44_ was reported^44^, relevant theoretical simulations indicated that neutral cage cluster B_44_ containing two nonagonal, two hexagonal and two heptagonal holes is the most stable structure among the isomers of B_44_. In addition, energies of first five lowest-lying isomers are close to each other, it is possible to expect that the five isomers may appear in the future experiments. It is necessary to study the structures and spectral characteristics of anionic B_44_^−^ and metalloborospherenes MB_44_^0/−^ (M = Li, Na, and K). The structure search algorithms and DFT combined approaches have been used and the low-lying structures of boron clusters have been reported by many authors^26^,^28^,^29^,^30^,^44^. It is not our purpose in this work to carry out an extensive structure search for the global minimum of B_44_^−^ cluster and metalloborospherenes MB_44_^0/−^ (M = Li, Na, and K). Instead, we will collect the B_44_ structures from the paper (*Chem. Commun*., 2016, 52, 1653–1656) and study the structures, stabilities of corresponding anionic B_44_^−^ and metalloborospherenes MB_44_^0/−^ (M = Li, Na, and K). Current works are therefore to provide a theoretical study on the stabilities, photoelectron spectra, infrared, Raman and electronic absorption spectra of B_44_^−^ and metalloborospherenes MB_44_^0/−^ (M = Li, Na, and K). Our works may provide valuable results to assist further experimental identifications on the borospherene B_44_^−^ and metalloborospherenes MB_44_^0/−^ (M = Li, Na, and K), and also may provide theoretical guidance for the applications and synthesis of them in the future.

To obtain the adiabatic detachment energy (ADE) and contrastive analysis, the five lowest-lying neutral isomers of B_44_ reported by Tai *et al*.^44^ were re-optimized using the density functional method PBE0 with 6–311 + G* basis set. The five corresponding anionic isomers were optimized using the DFT functionals TPSSh and PBE0 in conjunction with the 6–311 + G* basis set. To obtain more accurate relative energies, single-point electronic energies of the five anionic isomers were subsequently calculated using the coupled-cluster theory UCCSD(T)/3–21 G method at their PBE0/6–311 + G* optimized geometries. All ground-state geometries of the metalloborospherenes MB_44_^0/−^ and frequency calculations were performed based on the density functional method PBE0 with 6–311 + G* basis set. These optimized structures were then used in the calculations of photoelectron spectra and electronic absorption spectra based on the time-dependent DFT formalism^45^ at the same level. All computations were carried out using the Gaussian09 software package^46^.

## Results and Discussion

**Figure 1:**
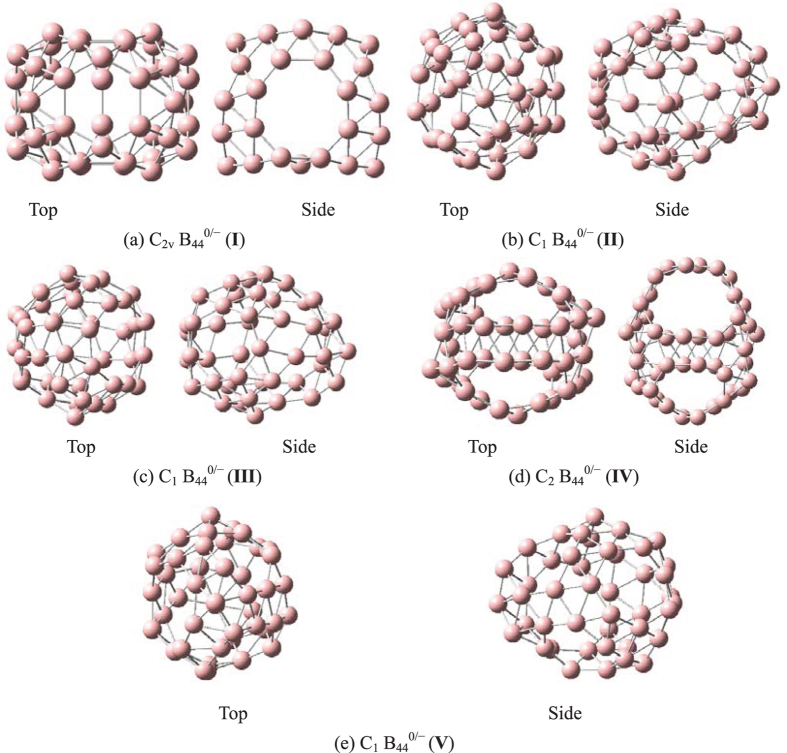
Optimized Structures for the five isomers of B_44_^0/−^ at the PBE0/6–311 + G* level.

Optimized structures of borospherenes B_44_^0/−^ are depicted in Fig. 1. Ground-state parameters are summarized in Table 1. Frequency calculations confirm the stability of B_44_^0/−^ by showing no imaginary frequencies. The relative energy values of neutral B_44_ agree well with the results of Tai *et al*.^44^ that isomer **IV** is the most stable form and isomer **I** is the third stable form. However, our TPSSh, PBE0 and UCCSD(T) energy values of anionic B_44_^−^ indicate that isomer **I** is the most stable form of the five isomers. Although the neutral isomer **II** and isomer **III** have different structures^44^ (isomer **II** includes two octagonal B_8_, two heptagonal B_7_ and two hexagonal B_6_ holes, isomer **III** includes two octagonal B_8_, three heptagonal B_7_ and one hexagonal B_6_ holes), optimized structures of anionic isomer **II** and **III** show that the two isomers have almost the same structure (two octagonal B_8_, two heptagonal B_7_ and two hexagonal B_6_ holes). It suggests that the two neutral isomers have the similar structures (only have a very small difference). In addition, for the sake of contrastive analysis, we also study the dianion B_44_^2−^ (**I** and **IV)**, ground-state parameters are summarized in Table S1. The relative energy values of dianion B_44_^2−^ indicate that B_44_^2−^ (**I**) is more stable than B_44_^2−^ (**IV**). Interestingly, isomer **I** is quite similar to cage B_40_ and includes two octagonal, four heptagonal and one hexagonal holes, it can be constructed by replacing two opposite heptagonal holes of the borospherene B_40_ by two octagonal holes and splitting one hexagonal hole of the B_40_ into two neighbouring heptagonal holes. We will focus on the two isomers (**I** and **IV)** and corresponding metalloborospherenes MB_44_^0/−^ (M = Li, Na, and K).

Optimized structures of metalloborospherenes MB_44_^0/−^ (M = Li, Na, and K) are depicted in Figure S1 (Supplementary Information). Ground-state parameters are summarized in Table 2. Frequency calculations confirm the stability of these endohedral and exohedral metalloborospherenes (except for endohedral Li@B_44_^0/−^) by showing no imaginary frequencies. Endohedral Li@B_44_^0/−^ (**I**) and Li@B_44_^0/−^ (**IV**) prove to be unstable with imaginary frequencies. The calculated results indicate that Na atoms in Na@B_44_^−^ (**I**), Na@B_44_ (**I**), Na@B_44_^−^ (**IV**) and Na@B_44_ (**IV**) are slightly off the molecular center by 0.14, 0.11, 1.28 and 1.18 Å, respectively, along the C_2_ molecular axis. In addition, K atoms in K@B_44_^−^ (**I**), K@B_44_ (**I**), K@B_44_^−^ (**IV**) and K@B_44_ (**IV**) are slightly off the molecular center by 0.12, 0.11, 0.09 and 0.04 Å, respectively, along the C_2_ molecular axis. Energies of these metalloborospherenes MB_44_^0/−^ (M = Na and K) indicate that most endohedral metalloborospherenes M@B_44_^0/−^ (M = Na and K) are less stable than corresponding exohedral metalloborospherenes M&B_44_^0/−^ (M = Na and K), respectively, whereas only the exohedral Na&B_44_^−^ (**IV**) is less stable than endohedral Na@B_44_^−^ (**IV**). The results reveal that Li, Na and K atoms favor the exohedral configuration. It’s worth noting that the energy differences between the endohedral metalloborospherenes M@B_44_^0/−^ (M = Na, K) and corresponding exohedral metalloborospherenes M&B_44_^0/−^ (M = Na, K) are small. Interestingly and encouragingly, although B_44_ (**I**) is less stable than B_44_ (**IV**), Table 2 shows that Li&B_44_ (**IV**), Na&B_44_ (**IV**), Na@B_44_ (**IV**), K&B_44_ (**IV**) and K@B_44_ (**IV**) are less stable than corresponding Li&B_44_ (**I**), Na&B_44_ (**I**), Na@B_44_ (**I**), K&B_44_ (**I**) and K@B_44_ (**I**), respectively, the addition of metal atom enhances the stability of isomer **I** compared with isomer **IV**. The results may provide a possible route (doping of metal atoms) to produce stable borospherenes or metalloborospherenes which have good properties and potential applications.

Photoelectron spectroscopy is powerful experimental technique to probe the electronic structure of cluster. It can be viewed as an electronic fingerprint for the underlying cluster. Photoelectron spectroscopy in combination with theoretical calculations has been used to understand and identify the structures of size-selected boron clusters^29^,^30^. To facilitate future identifications of B_44_^−^, the ADEs for B_44_^−^ and metalloborospherenes MB_44_^−^ (M = Li, Na, and K) were calculated at the PBE0 level, then we calculated the vertical detachment energies (VDEs) and simulated the photoelectron spectra for B_44_^−^ and metalloborospherenes MB_44_^−^ (M = Li, Na, and K), using the time-dependent DFT (TD-DFT) method^29^,^30^,^45^. Adiabatic detachment energy of B_44_^−^ represents the electron affinity (EA) of corresponding neutral B_44_. The larger EA can lead to the stronger probability of capturing an electron, i.e., the neutral B_44_ with larger EA is easier to capture an electron. The five isomers give the ground-state ADEs of 3.23(**I**), 3.02(**II**), 3.02(**III**), 2.78(**IV**) and 2.99(**V**) eV, respectively. Among the five isomers of B_44_^−^, isomer **I** has the largest ADE (3.23 eV), which is larger than the ADE (2.29 eV)^29^ of cage B_40_^−^ and less than the ADE (3.51 eV)^29^ of quasi-planar B_40_^−^. The calculated ground-state ADEs of Li&B_44_^−^ (**I**), Li&B_44_^−^ (**IV**), Na&B_44_^−^ (**I**), Na&B_44_^−^ (**IV**), Na@B_44_^−^ (**I**), Na@B_44_^−^ (**IV**), K&B_44_^−^ (**I**), K&B_44_^−^ (**IV**), K@B_44_^−^ (**I**) and K@B_44_^−^ (**IV**) are 3.17, 2.96, 3.01, 2.79, 3.24, 3.11, 2.87, 2.63, 3.22 and 3.01 eV, respectively. The calculated results indicate that doping of alkali metal atom in B_44_^−^ (**I**) can decrease the ADE of B_44_^−^ (**I**), however, doping of alkali metal atom in B_44_^−^ (**IV**) can increase the ADE of B_44_^−^ (**IV**).

**Figure 2:**
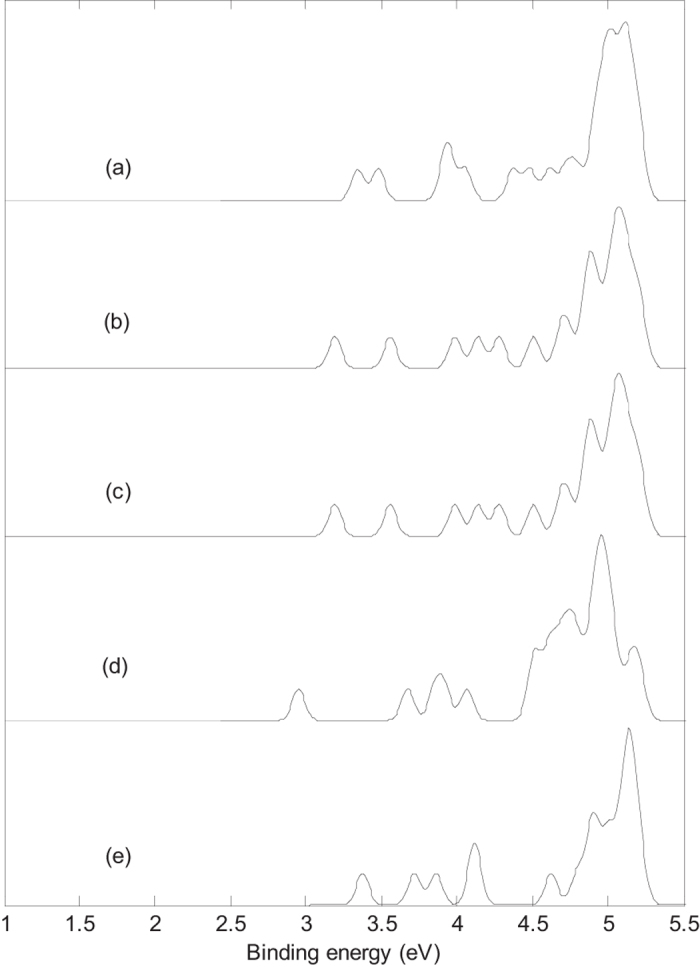
Simulated photoelectron spectra for the 5 isomers of B_44_^−^ based on PBE0 functional with 6–311 + G* basis set. (**a**): C_2v_ B_44_^−^ (**I**), (**b**): C_1_ B_44_^−^ (**II**), (**c**): C_1_ B_44_^−^ (**III**), (**d**): C_2_ B_44_^−^ (**IV**), (**e**): C_1_ B_44_^−^ (**V**). The simulations were done by fitting the distributions of calculated vertical detachment energies at the PBE0 level with unit-area Gaussian functions of 0.05 eV half-width.

Photoelectron spectra of five isomers are given in Fig. 2. The predicted photoelectron spectra show that isomer **IV** has the lowest first vertical detachment energy (VDE) and the largest energy gap (about 0.73 eV) between the first and second bands. The first several bands of photoelectron spectra were used to identify boron clusters^29^,^30^, so we will focus on the bands at the low binding energy side. The first peaks of five isomers come from the calculated ground-state VDEs at 3.34(**I**), 3.19(**II**), 3.19(**III**), 2.95(**IV**) and 3.38(**V**) eV, respectively. The calculated ground-state VDE of each isomer originates from the detachment of the electron from the singly occupied molecular orbital (*α*-SOMO). The second peaks of the five isomers come from the second calculated VDEs at 3.48(**I**), 3.56(**II**), 3.56(**III**), 3.68(**IV**) and 3.72(**V**) eV, respectively. The second calculated VDEs of five isomers originate from detaching the electron from β-HOMO-1 resulting in the first triplet state. Figure 2(b,c) indicate that isomer **II** and isomer **III** have the same photoelectron spectrum, which confirms that the two isomers have almost the same structure.

**Figure 3:**
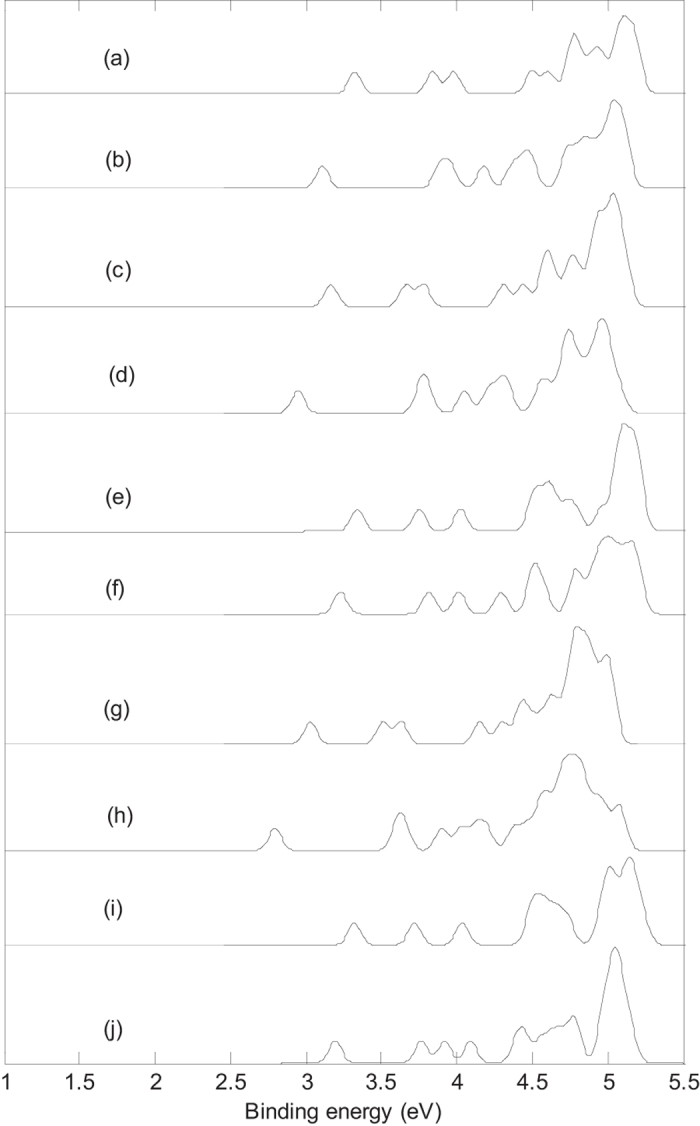
Simulated photoelectron spectra of metalloborospherenes MB_44_^−^ (M = Li, Na, and K) based on PBE0 functional with 6–311 + G* basis set. (**a**): C_s_ Li&B_44_^−^ (**I**), (**b**): C_1_ Li&B_44_^−^ (**IV)**, (**c**): C_s_ Na&B_44_^−^ (**I**), (**d**): C_1_ Na&B_44_^−^ (**IV)**, (**e**): C_2v_ Na@B_44_^−^ (**I**), (**f**): C_2_ Na@B_44_^−^ (**IV)**, (**g**): C_s_ K&B_44_^−^ (**I**), (**h**): C_1_ K&B_44_^−^ (**IV)**, (**i**): C_2v_ K@B_44_^−^ (**I**), (**j**): C_2_ K@B_44_^−^ (**IV)**. The simulations were done by fitting the distributions of calculated vertical detachment energies at the PBE0 level with unit-area Gaussian functions of 0.05 eV half-width.

Figure 3 presents the photoelectron spectra of metalloborospherenes MB_44_^−^ (M = Li, Na, and K). The first peaks of the photoelectron spectra come from the calculated ground-state VDEs of Li&B_44_^−^ (**I**), Li&B_44_^−^ (**IV**), Na&B_44_^−^ (**I**), Na&B_44_^−^ (**IV**), Na@B_44_^−^ (**I**), Na@B_44_^−^ (**IV**), K&B_44_^−^ (**I**), K&B_44_^−^ (**IV**), K@B_44_^−^ (**I**) and K@B_44_^−^ (**IV**) at 3.32, 3.11, 3.17, 2.95, 3.34, 3.23, 3.03, 2.70, 3.32 and 3.15 eV, respectively. The calculated ground-state VDEs of metalloborospherenes originate from the detachment of the electron from the molecular orbital (HOMO). The predicted photoelectron spectra in Fig. 3 show that the first VDEs of Li&B_44_^−^ (**I**), Na&B_44_^−^ (**I**), K&B_44_^−^ (**I**), Na@B_44_^−^ (**I**) and K@B_44_^−^ (**I**) are lager than that of Li&B_44_^−^ (**IV**), Na&B_44_^−^ (**IV**), K&B_44_^−^ (**IV**), Na@B_44_^−^ (**IV**) and K@B_44_^−^ (**IV**), respectively. The second peaks of Li&B_44_^−^ (**I**), Na&B_44_^−^ (**I**), Na@B_44_^−^ (**I**), Na@B_44_^−^ (**IV**), K&B_44_^−^ (**I**), K@B_44_^−^ (**I**) and K@B_44_^−^ (**IV**) come from the second calculated VDEs at 3.84, 3.66, 3.75, 3.82, 3.51, 3.72 and 3.76 eV, respectively. The second calculated VDEs of metalloborospherenes originate from detaching the electron from HOMO-1. The second peaks of Li&B_44_^−^ (**IV**), Na&B_44_^−^ (**IV**) and K&B_44_^−^ (**IV**) come from the second and third calculated VDEs, interestingly, the second and third calculated VDEs of each metalloborospherene (Li&B_44_^−^ (**IV**), Na&B_44_^−^ (**IV**) or K&B_44_^−^ (**IV**)) are almost overlapped and correspond to an electron detachment from the HOMO-1 and HOMO-2 (orbital energies of HOMO-1 and HOMO-2 are almost degenerate). Figure 3(a,c,g) indicate that exohedral Li&B_44_^−^ (**I**), Na&B_44_^−^ (**I**) and K&B_44_^−^ (**I**) have the similar spectral features, however, all bands move to lower binding energy side with the increase of radius of doped atom. Similarly, exohedral Li&B_44_^−^ (**IV**), Na&B_44_^−^ (**IV**) and K&B_44_^−^ (**IV**) also have such characteristics. The simulated photoelectron spectrum of Na@B_44_^−^ (**IV**) is somewhat similar to the spectrum of Na@B_44_^−^ (**I**), whereas the third peak of Na@B_44_^−^ (**IV**) can be used to distinguish the Na@B_44_^−^ (**I**) and Na@B_44_^−^ (**IV**). Figure 3(e,i) indicate that simulated photoelectron spectra of Na@B_44_^−^ (**I**) and K@B_44_^−^ (**I**) have the similar spectral features, particularly, the first three peaks are almost same. Figure 3 indicate that photoelectron spectra of Li&B_44_^−^ (**I**), Na&B_44_^−^ (**I**), K&B_44_^−^ (**I**), Na@B_44_^−^ (**I**) and K@B_44_^−^ (**I**) are different from that of Li&B_44_^−^ (**IV**), Na&B_44_^−^ (**IV**), K&B_44_^−^ (**IV**), Na@B_44_^−^ (**IV**) and K@B_44_^−^ (**IV**), respectively. These features can be used to distinguish the metalloborospherenes MB_44_^−^ (**I**) and metalloborospherenes MB_44_^−^ (**IV**).

Figures 2(a,d) and 3 indicate that the addition of alkali metal atom modifies the photoelectron spectra of B_44_^−^ since the addition of extra atom modifies the electronic structure. The calculated results indicate that doping of alkali metal atom in B_44_^−^ (**I**) can decrease the first VDE of B_44_^−^ (**I**), however, doping of alkali metal atom in B_44_^−^ (**IV**) can increase the first VDE of B_44_^−^ (**IV**). The predicted photoelectron spectra in Figs 2 and 3 show that B_44_^−^ and metalloborospherenes MB_44_^−^ (M = Li, Na, and K) have different spectral features, the predicted photoelectron spectra provide important information for the identification of B_44_^−^ and metalloborospherenes MB_44_^−^ (M = Li, Na, and K). It is worth to note that the structures of atomic clusters cannot directly be identified by common analytical expermenttal methods, but they can indirectly be determined by using combined theoretical and experimental studies. As the discovery of B_40_, if the photoelectron spectra of B_44_^−^ and metalloborospherenes MB_44_^−^ (M = Li, Na, and K) are obtained in experiments, these calculated characteristic bands may be used as theoretical basis for the identification of boron cluster B_44_^−^ and metalloborospherenes MB_44_^−^ (M = Li, Na, and K).

**Figure 4:**
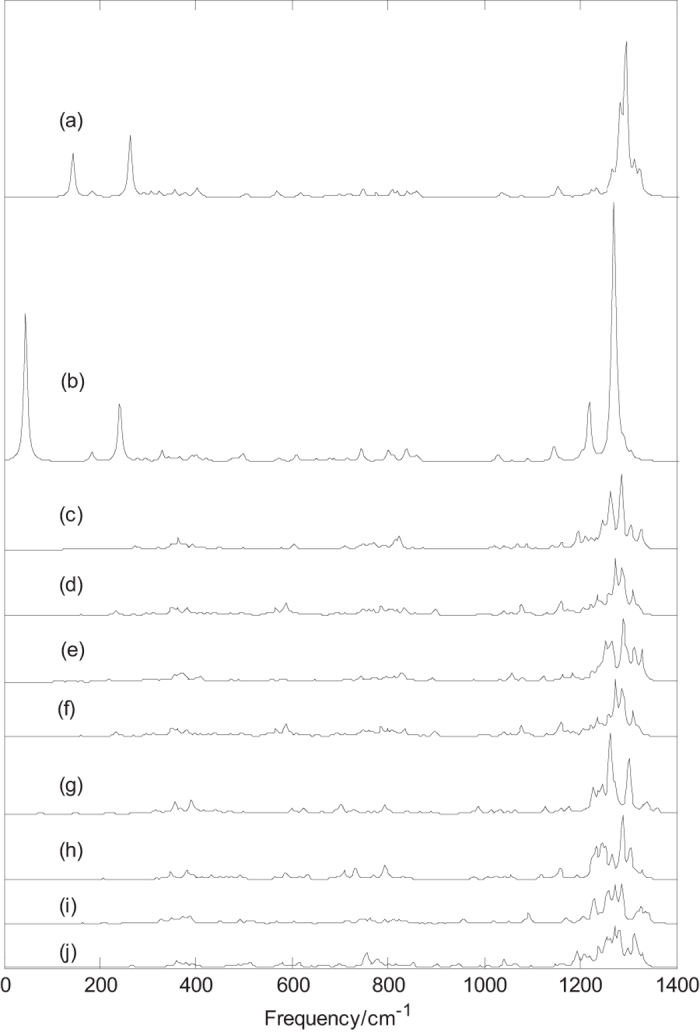
Predicted infrared spectra of B_44_^0/−^ based on PBE0 functional with 6–311 + G* basis set. (**a**): C_2v_ B_44_ (**I**), (**b**):C_2v_ B_44_^−^ (**I**), (**c**): C_1_ B_44_ (**II**), (**d**): C_1_ B_44_^−^ (**II**), (**e**): C_1_ B_44_ (**III**), (**f**): C_1_ B_44_^−^ (**III**), (**g**): C_2_ B_44_ (**IV)**, (**h**): C_2_ B_44_^−^ (**IV)**, (**i**): C_1_ B_44_ (**V)**, (**j**): C_1_ B_44_^−^ (**V)**.

Normal mode frequencies, infrared intensities and Raman activities of B_44_^0/−^ and metalloborospherenes MB_44_^0/−^ (M = Li, Na, and K) are calculated and depicted in Figs 4 and 7. Predicted spectral peaks distribute in three regions: low frequency region (from 0 to 600 cm^−1^), middle frequency region (from 600 to 1000 cm^−1^) and high frequency region (from 1000 to 1600 cm^−1^). These vibrational modes within high frequency region are closely related to molecular structure. This suggests that molecular with slightly difference can lead to the subtle differences of infrared absorption in this region, namely, the infrared spectra of molecular show the characteristics of molecular, like fingerprints, known as fingerprint region.

Infrared spectra of borospherenes B_44_^0/−^ are given in Fig. 4. Figure 4(a) presents the infrared spectrum of B_44_ (**I**), the sharpest peak occurs at 1295 cm^−1^. In addition, at 143 and 262 cm^−1^, the characteristic peaks are strong, which is different from other isomers. The two strong peaks are produced by bending vibration of boron atoms and they belong to the far-infrared region. It’s worth noting that infrared spectrum of B_44_ (**I**) is somewhat similar to that of borospherene B_40_ except for the two peaks at 143 and 262 cm^−1^. Figure 4(b) presents the infrared spectrum of B_44_^−^ (**I**), the sharpest peak occurs at 1271 cm^−1^. The computed IR spectra of B_44_^0/−^ (**I**) indicate that there are some IR inactive modes and only a few of IR active modes have strong absorption. As shown in Fig. 4(a,b), the addition of an electron does not change the symmetry, but leads to an other strong peaks (at 1217 cm^−1^) in the high frequency region and redshifts the three main peaks from 1295, 262 and 143 cm^−1^ for B_44_ (**I**) to 1271, 241, and 45 cm^−1^ for B_44_^−^ (**I**), respectively, which will be useful to identify the anionic B_44_^−^ (**I**) and neutral B_44_ (**I**). Figure 4(c–j) present the infrared spectra of B_44_^0/−^ (**II-V**), the sharpest peaks of B_44_ (**II**), B_44_^−^ (**II**), B_44_ (**III**), B_44_^−^ (**III**), B_44_ (**IV**), B_44_^−^ (**IV**), B_44_ (**V**) and B_44_^−^ (**V**) are at 1285, 1274, 1289, 1274, 1262, 1287, 1286 and 1281 cm^−1^, respectively. The sharpest peaks of these borospherenes B_44_^0/−^ are at high frequency region. Figure 4(d,f) show that B_44_^−^ (**II**) and B_44_^−^ (**III**) have almost the same infrared spectrum, however, Fig. 4(c,e) show that B_44_ (**II)** and B_44_ (**III**) have the similar infrared spectra, which further indicates that the two neutral isomers have the similar structures, instead of same structure. The B_44_ (**IV**) has two strong characteristic peaks at 1262 and 1301 cm^−1^, whereas the addition of an electron weakens the two strong vibrational modes and leads to another strong characteristic peak at 1287 cm^−1^. These features can be used to distinguish the B_44_ (**IV**) and B_44_^−^ (**IV**). Figure 4(a,b,g,h) show that B_44_^0/−^ (**I**) and B_44_^0/−^ (**IV**) have different characteristic peaks, which can be used to distinguish B_44_^0/−^ (**I**) and B_44_^0/−^ (**IV**).

**Figure 5:**
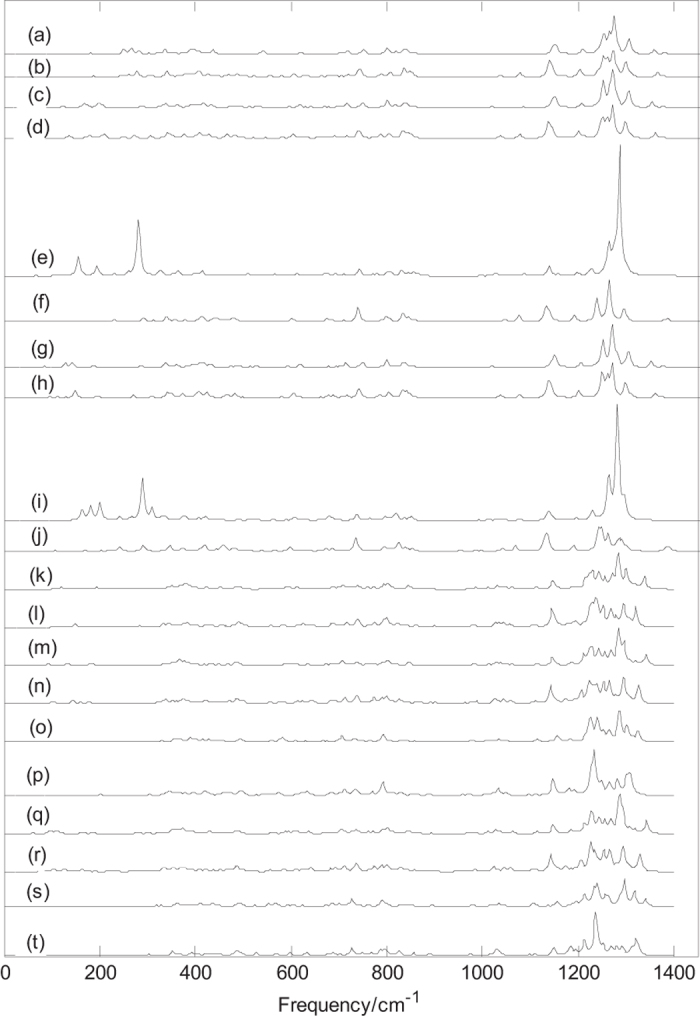
Predicted infrared spectra of metalloborospherenes MB_44_^0/−^ (M = Li, Na, and K) based on PBE0 functional with 6–311 + G* basis set. (**a**): C_s_ Li&B_44_ (**I**), (**b**):C_s_ Li&B_44_^−^ (**I**), (**c**): C_s_ Na&B_44_ (**I**), (**d**): C_s_ Na&B_44_^−^ (**I**), (**e**): C_2v_ Na@B_44_ (**I**), (**f**): C_2v_ Na@B_44_^−^ (**I**), (**g**): C_s_ K&B_44_ (**I**), (**h**): C_s_ K&B_44_^−^ (**I**), (**i**): C_2v_ K@B_44_ (**I**), (**j**): C_2v_ K@B_44_^−^ (**I**), (**k**): C_1_ Li&B_44_ (**IV**), (**l**):C_1_ Li&B_44_^−^ (**IV**), (**m**): C_1_ Na&B_44_ (**IV**), (**n**): C_1_ Na&B_44_^−^ (**IV**), (**o**): C_2_ Na@B_44_ (**IV**), (**p**): C_2_ Na@B_44_^−^ (**IV**), (**q**): C_1_ K&B_44_ (**IV**), (**r**): C_1_ K&B_44_^−^ (**IV**), (**s**): C_2_ K@B_44_ (**IV**), (**t**): C_2_ K@B_44_^−^ (**IV**).

Figure 5 shows that the most strong infrared peaks of metalloborospherenes MB_44_^0/−^ (M = Li, Na, and K) distribute in high-frequency region (from 1000 to 1400 cm^−1^), and other peaks are relatively weak. Figure 5(a–j) present the infrared spectra of M&B_44_^0/−^ (**I**), the sharpest peaks of Li&B_44_ (**I**), Li&B_44_^−^ (**I**), Na&B_44_ (**I**), Na&B_44_^−^ (**I**), Na@B_44_ (**I**), Na@B_44_^−^ (**I**), K&B_44_ (**I**), K&B_44_^−^ (**I**), K@B_44_ (**I**) and K@B_44_^−^ (**I**) are at 1274, 1272, 1272, 1271, 1286, 1264, 1270, 1270, 1281 and 1241 cm^−1^, respectively. The sharpest peaks of metalloborospherenes MB_44_^0/−^ (M = Li, Na, and K) are at high frequency region, and these vibrational modes formed by stretching vibrations of boron atoms. Figure 5(a–d,g,h) show that exohedral M&B_44_^0/−^ (**I**, M = Li, Na, and K) have similar infrared spectra, but the main peaks of each anionic metalloborospherene are redshifted somewhat since the extra electron modifies the electronic structures. Figure 5(e,f,i,j) present the infrared spectra of endohedral M@B_44_^0/−^ (**I**, M = Na, and K), and the results show that the addition of an electron weakens some strong vibrational modes and leads to some strong characteristic peaks. Figure 5(k–t) present the infrared spectra of M&B_44_^0/−^ (**IV**), the sharpest peaks of Li&B_44_ (**IV**), Li&B_44_^−^ (**IV**), Na&B_44_ (**IV**), Na&B_44_^−^ (**IV**), Na@B_44_ (**IV**), Na@B_44_^−^ (**IV**), K&B_44_ (**IV**), K&B_44_^−^ (**IV**), K@B_44_ (**IV**) and K@B_44_^−^ (**IV**) are at 1284, 1294, 1285, 1294, 1286, 1234, 1287, 1226, 1297 and 1236 cm^−1^, respectively. Like the exohedral M&B_44_ (**I**), Fig. 5(k–n,q,r) show that exohedral M&B_44_ (**IV**) have the similar infrared spectra. The predicted infrared spectra in Fig. 5 show that metalloborospherenes M&B_44_^0/−^ (**IV**) have different spectral features and characteristic peaks compared with the corresponding M&B_44_^0/−^ (**I**) such as M&B_44_^0/−^ (**I**) contain some vibration modes with higher frequencies.

Infrared spectra of B_44_^0/−^ (**I**) are different from that of exohedral M&B_44_^0/−^ (**I**, M = Li, Na, and K), the metal dopant in B_44_^0/−^ (**I**) changes the IR spectra of B_44_^0/−^ (**I**) such as some weakened vibrational modes and some enhanced characteristic peaks. Note that the sharpest peaks of exohedral M&B_44_^0/−^ (**I**, M = Li, Na, and K) are located at about 1270 cm^−1^ and are almost same with the location (at 1271 cm^−1^) of sharpest peak for B_44_^−^ (**I**), but the intensity of sharpest peaks for M&B_44_^0/−^ (**I**, M = Li, Na, and K) are significantly weakened. Figures 4(a) and 5(e,i) show that the infrared spectra of endohedral Na@B_44_ and K@B_44_ are similar to that of B_44_, whereas the addition of metal atom weakens some strong vibrational modes and leads to some strong characteristic peaks. It’s worth noting that the infrared spectra of exohedral M&B_44_^0/−^ (**I**, M = Li, Na, and K) and endohedral M@B_44_^−^ (**I**, M = Na, and K) are quite similar to that of dianion B_44_^2−^ (**I**)(Figure S2). It suggests that B_44_ (**I**) tends to get electrons from the extra electron and the doped metal. As the analysis of M@B_40_ (M = Ca,Sr) and M&B_40_ (M = Be,Mg)^35^, exohedral metalloborospherenes M&B_44_^0/−^ (**I**, M = Li, Na, and K) and endohedral M@B_44_^−^ (**I**, M = Na, and K) are characterized as charge-transfer complexes (M^2+^B_44_^2−^), where metal atom donates one electron or two electrons to B_44_ (**I**), resulting in similar features with anionic B_44_^2−^ (**I**). Similarly, infrared spectra of exohedral M&B_44_^−^ (**IV**, M = Li, Na, and K) and endohedral M@B_44_^−^ (**IV**, M = Na, and K) are somewhat similar to that of dianion B_44_^2−^ (**IV**) (Figure S2). It suggests that the extra electron moves to B_44_ (**IV**) and the doped metal donates one electron to B_44_ (**IV**), resulting in similar features with anionic B_44_^2−^(**IV**). However, exohedral M&B_44_ (**IV**, M = Li, Na, and K) and endohedral M@B_44_ (**IV**, M = Na, and K) are somewhat similar to that of anion B_44_^−^ (**IV**). It suggests that B_44_ (**IV**) tends to get one electron from the doped metal. The predicted infrared spectra also provide some information for the identification of B_44_^−^ and metalloborospherenes MB_44_^−^ (M = Li, Na, and K), these different characteristic peaks provide a theoretical basis for the identification and confirmation of B_44_^−^ and metalloborospherenes MB_44_^0/−^ (M = Li, Na, and K).

**Figure 6:**
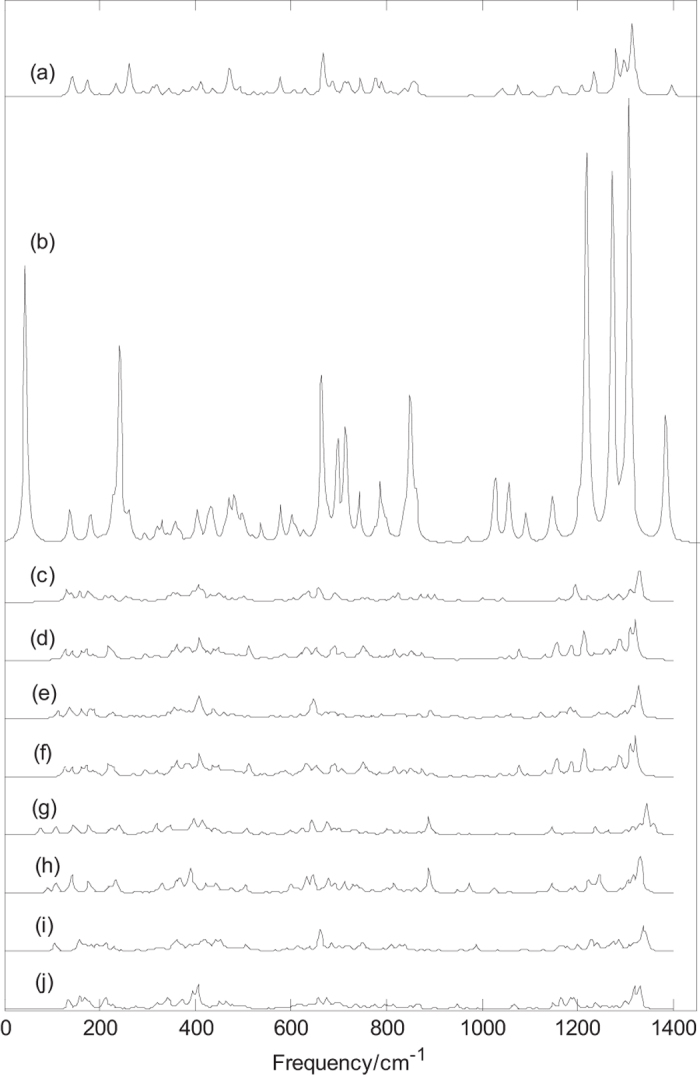
Predicted Raman spectra for the 5 isomers of B_44_^0/−^ based on PBE0 functional with 6–311 + G* basis set. (**a**): C_2v_ B_44_ (**I**), (**b**): C_2v_ B_44_^−^ (**I**), (**c**): C_1_ B_44_ (**II**), (**d**): C_1_ B_44_^−^ (**II**), (**e**): C_1_ B_44_ (**III**), (**f**): C_1_ B_44_^−^ (**III**), (**g**): C_2_ B_44_ (**IV**), (**h**): C_2_ B_44_^−^ (**IV**), (**i**): C_1_ B_44_ (**V**), (**j**): C_1_ B_44_^−^ (**V**).

Figure 6 depicts the Raman spectra of B_44_^0/−^. Figure 6(a) depicts the Raman spectrum of B_44_ (**I**), the sharpest peak occurs at 1312 cm^−1^. Among the Raman active modes, the vibration at 144 cm^−1^ belongs to typical radial breathing mode, which is similar to the typical radial breathing mode of B_40_^47^ at 170 cm^−1^. The breathing modes are used to identify the hollow structures in nanotubes. Figure 6(b) depicts the Raman spectrum of B_44_^−^ (**I**), the sharpest peak occurs at 1307 cm^−1^. Similar to B_44_ (**I**), the vibration at 139 cm^−1^ belongs to typical radial breathing mode, which is similar to the typical radial breathing mode of B_40_^−^ 43 at 176 cm^−1^. Figure 6(b) indicates that there are four main Raman peaks in the high frequency region and some strong Raman peaks in the middle and lower frequency regions. It’s worth noting that the Raman spectrum of B_44_^−^ (**I**) is far stronger than that of other B_44_^0/−^. The computed Raman spectra of B_44_^0/−^ (**I**) suggest that all vibration modes are Raman active modes, but only a few of them have strong Raman activity. Figure 6(c–j) depict the Raman spectra of B_44_^0/−^ (**II-V**), the sharpest peaks for B_44_ (**II**), B_44_^−^ (**II**), B_44_ (**III**), B_44_^−^ (**III**), B_44_ (**IV**), B_44_^−^ (**IV**), B_44_ (**V**) and B_44_^−^ (**V**) are at 1329, 1321, 1327, 1321, 1344, 1328, 1335 and 1329 cm^−1^, respectively. The sharpest Raman peaks of B_44_^0/−^ are at high frequency region, and these vibrational modes are formed by stretching vibration of boron atoms. Figure 6(d,f) show that B_44_^−^ (**II**) and B_44_^−^ (**III**) have almost the same Raman spectrum, however, Fig. 6(c,e) show that B_44_ (**II**) and B_44_ (**III**) have the similar Raman spectra. It further indicates that the two neutral isomers have the similar structures and the two anionic isomers have almost the same structure. Figure 6 indicates that the addition of an electron leads to the redshift of sharpest peak for each isomer. In addition, the calculated results indicate that all vibrational modes of B_44_^0/−^ (**II**), B_44_^0/−^ (**III**), B_44_^0/−^ (**IV**) and B_44_^0/−^ (**V**) are infrared active and Raman active, however, some vibrational modes of B_44_^0/−^ (**I**) are infrared inactive. The relatively high symmetric structure (C_2v_) of B_44_^0/−^ (**I**) may lead to the infrared inactive vibrational modes.

**Figure 7:**
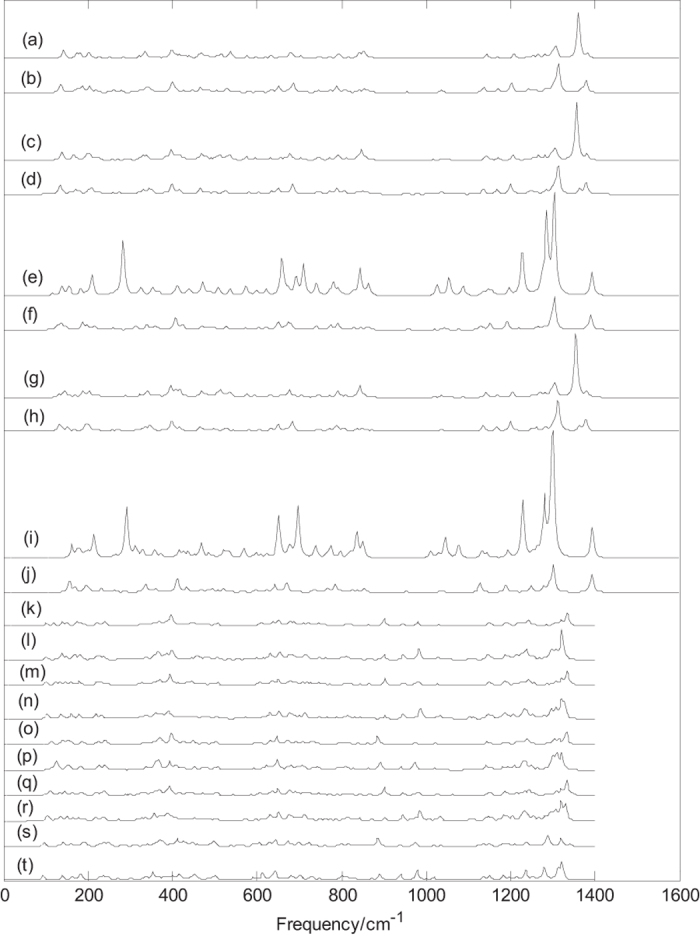
Predicted Raman spectra of metalloborospherenes MB_44_^0/−^ (M = Li, Na, and K) based on PBE0 functional with 6–311 + G* basis set. (**a**): C_s_ Li&B_44_ (**I**), (**b**): C_s_ Li&B_44_^−^ (**I**), (**c**): C_s_ Na&B_44_ (**I**), (**d**): C_s_ Na&B_44_^−^ (**I**), (**e**): C_2v_ Na@B_44_ (**I**), (**f**): C_2v_ Na@B_44_^−^ (**I**), (**g**): C_s_ K&B_44_ (**I**), (**h**): C_s_ K&B_44_^−^ (**I**), (**i**): C_2v_ K@B_44_ (**I**), (**j**): C_2v_ K@B_44_^−^ (**I**), (**k**): C_1_ LiB_44_ (**IV**), (**l**): C_1_ LiB_44_^−^ (**IV**), (**m**): C_1_ Na&B_44_ (**IV**), (**n**): C_1_ Na&B_44_^−^ (**IV**), (**o**): C_2_ Na@B_44_ (**IV**), (**p**): C_2_ Na@B_44_^−^ (**IV**), (**q**): C_1_ K&B_44_ (**IV**), (**r**): C_1_ K&B_44_^−^ (**IV**), (**s**): C_2_ K@B_44_ (**IV**), (**t**): C_2_ K@B_44_^−^ (**IV**).

Figure 7 depicts the Raman spectra of metalloborospherenes MB_44_^0/−^ (M = Li, Na, and K). Figure 7(a–j) depict the Raman spectra of M&B_44_ (**I**, M = Li, Na, and K), the sharpest peaks for Li&B_44_ (**I**), Li&B_44_^−^ (**I**), Na&B_44_ (**I**), Na&B_44_^−^ (**I**), Na@B_44_ (**I**), Na@B_44_^−^ (**I**), K&B_44_ (**I**), K&B_44_^−^ (**I**), K@B_44_ (**I**) and K@B_44_^−^ (**I**) are at 1361, 1314, 1356, 1314, 1305, 1305, 1355, 1313, 1302 and 1303 cm^−1^, respectively. The calculated results indicate that all vibrational modes of Li&B_44_^0/−^ (**I**), Na&B_44_^0/−^ (**I**) and K&B_44_ (**I**) are infrared active and Raman active, however, some vibrational modes of K&B_44_^−^ (**I**), Na@B_44_^0/−^ (**I**) and K@B_44_^0/−^ (**I**) are infrared inactive. Figure 7(a–d,g,h) depict the Raman spectra of exohedral M&B_44_^0/−^ (**I**, M = Li, Na, K), they have the similar spectral features. Figure 7(a,c,g) show that exohedral M&B_44_ (**I**, M = Li, Na, and K) have almost the same Raman spectrum, and Fig. 7(b,d,h) show that exohedral M&B_44_^−^ (**I**, M = Li, Na, and K) have almost the same Raman spectrum. Interestingly, the addition of an electron blueshifts the first two strong peaks and reverses the intensity of first two strong peaks. Figure 7(e,i) show that endohedral Na@B_44_ (**I**) and K@B_44_ (**I**) have the similar Raman spectra, and Fig. 7(f,j) show that endohedral Na@B_44_^−^ (**I**) and K@B_44_^−^ (**I**) have the similar Raman spectra. Figure 7(e,f,i,j) show that the addition of an electron weakens some strong characteristic peaks. Figure 7(k–t) depict the Raman spectra of M&B_44_ (**IV**, M = Li, Na, and K), the sharpest peaks of Li&B_44_ (**IV**), Li&B_44_^−^ (**IV**), Na&B_44_ (**IV**), Na&B_44_^−^ (**IV**), Na@B_44_ (**IV**), Na@B_44_^−^ (**IV**), K&B_44_ (**IV**), K&B_44_^−^ (**IV**), K@B_44_ (**IV**) and K@B_44_^−^ (**IV**) are at 1334, 1321, 1334, 1320, 1332, 1321, 1333, 1320, 1319 and 1321 cm^−1^, respectively. Figure 7(k–r) indicate that the addition of an electron leads to the redshift of sharpest peak.

Figures 6 and 7 indicate that doping of metal atom in B_44_^0/−^ (**I**, **IV**) changes the Raman peaks of B_44_^0/−^ (**I**, **IV**) such as some weakened vibrational modes and some enhanced characteristic peaks. It’s worth noting that the Raman spectra of exohedral M&B_44_^0/−^ (**I**, M = Li, Na, and K) and endohedral M@B_44_^−^ (**I**, M = Na, and K) are similar to that of dianion B_44_^2−^ (Figure S3). It further suggests that B_44_ (**I**) tends to get electrons from the extra electron and the doped metal. Exohedral metalloborospherenes M&B_44_^0/−^ (**I**, M = Li, Na, and K) and endohedral M@B_44_^−^ (**I**, M = Na, and K) are characterized as charge-transfer complexes (M^2+^B_44_^2−^), where metal atom donates one electron or two electrons to B_44_(**I**), resulting in similar features with anionic B_44_^2−^(**I**). Like the infrared spectra of metalloborospherenes M&B_44_^−^ (**IV**, M = Li, Na, and K), Raman spectra of exohedral M&B_44_^−^ (**IV**, M = Li, Na, and K) and endohedral M@B_44_^−^ (**IV**, M = Na, and K) are somewhat similar to that of dianion B_44_^2−^ (**IV**) (Figure S3), it further suggests that B_44_ (**IV**) tends to get two electrons from the extra electron and the doped metal, respectively. Raman spectra, as the supplement of infrared spectra, can also be used for the basis of identification of B_44_^0/−^ and metalloborospherenes MB_44_^0/−^ (M = Li, Na, and K). From the infrared and Raman spectra of each borospherene or metalloborospherene, we can find, at some frequencies, infrared absorption peaks are strong, but the Raman peaks are very weak. However, at some frequencies, the relation is just opposite. In addition, at some frequencies, both the infrared and Raman peaks are strong. A vibrational mode of molecular with no change of dipole moment is infrared inactive, we can’t obtain the normal mode frequency from the infrared spectral data in experiments. However, this vibrational mode may lead to the change of polarizability, this indicates that the vibrational mode is Raman active. The calculated Raman spectra can be useful for analytical purposes and contribute significantly to spectral interpretation and vibrational assignments, also can provide technical guidance for future synthesis.

**Figure 8:**
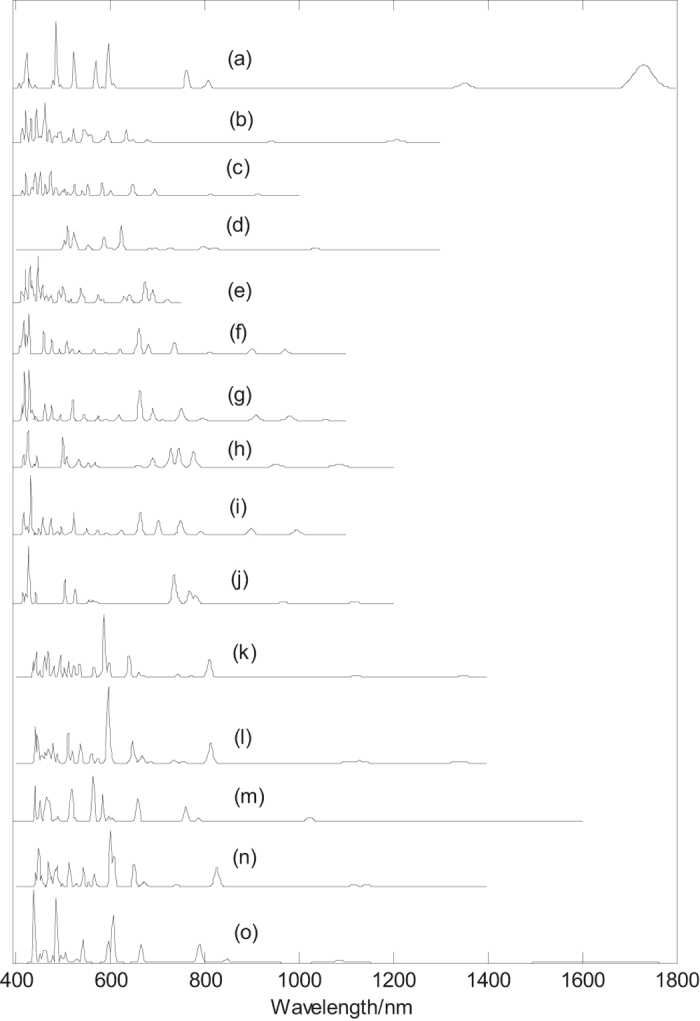
Predicted electronic absorption spectra of B_44_ and metalloborospherenes MB_44_^−^ (M = Li, Na, and K) with closed-shell electronic structure based on PBE0 functional with 6–311 + G* basis set. (**a**): C_2v_ B_44_ (**I**), (**b**): C_1_ B_44_ (**II**), (**c**): C_1_ B_44_ (**III**), (**d**): C_2_ B_44_ (**IV**), (**e**): C_1_ B_44_ (**V**), (**f**): C_s_ Li&B_44_^−^ (**I**), (**g**): C_s_ Na&B_44_^−^ (**I**), (**h**): C_2v_ Na@B_44_^−^ (**I**), (**i**): C_s_ K&B_44_^−^ (**I**), (**j**): C_2v_ K@B_44_^−^ (**I**), (**k**): C_1_ Li &B_44_^−^ (**IV**), (**l**): C_1_ Na&B_44_^−^ (**IV**), (**m**): C_2_ Na@B_44_^−^ (**IV**), (**n**): C_1_ K&B_44_^−^ (**IV**), (**o**): C_2_ K@B_44_^−^ (**IV**).

Finally, we calculated electronic absorption spectra of B_44_ and metalloborospherenes MB_44_^−^ (M = Li, Na, K) with closed-shell electronic structure, as shown in Fig. 8. Figure 8(a–e) present the electronic absorption spectra of B_44_ (**I**, **II**, **III**, **IV**, and **V**), the calculated strongest absorption peaks and the largest excitation wavelengths of B_44_ (**I**, **II**, **III**, **IV**, and **V**) are at 485, 460 472, 622 and 446 nm and 1730, 1207, 913 1247 and 720 nm, respectively. Note that the oscillator strength of largest excitation wavelength for isomer **IV** is zero, and the largest excitation wavelength (with small oscillator strength) is 1035 nm. The minimum excitation energy (the largest excitation wavelength) mainly comes from the electron transition from HOMO to LUMO. HOMO–LUMO energy gap reflects the probability of the molecules jumping from ground state to excited state. Generally speaking, the larger energy gap can lead to the larger electron excitation energy, i.e., the smaller the probability of electronic transition. On the contrary, the molecule with smaller energy gap is easier to jump to the excited state. According to the previous results, the HOMO–LUMO energy gaps are 1.35, 1.85, 2.12, 2.24 and 2.59 eV for B_44_ (**I**), B_44_ (**II**), B_44_ (**IV**), B_44_ (**III**) and B_44_ (**V**), respectively. Although the energy gap of ground state does not represent the minimum excitation energy, the increasing HOMO–LUMO energy gaps just reflect the decreasing largest excitation wavelengths 1730, 1207, 1035, 913, and 720 nm for B_44_ (**V**), B_44_(**III**), B_44_ (**IV**), B_44_(**II**) and B_44_ (**I**), respectively. Figure 8(a–e) indicate that electronic absorption spectrum of B_44_ (**I**) is apparently red-shifted comparing with other isomers.

Figure 8(f–j) present the electronic absorption spectra of metalloborospherenes MB_44_^−^ (**I**), the calculated largest excitation wavelengths of Li&B_44_^−^ (**I**), Na&B_44_^−^ (**I**), Na@B_44_^−^ (**I**), K&B_44_^−^ (**I**) and K@B_44_^−^ (**I**) are at 1048, 1057, 1112 1044 and 1118 nm, respectively. However, the oscillator strength of the largest excitation wavelengths of Li&B_44_^−^ (**I**), Na@B_44_^−^ (**I**) and K&B_44_^−^ (**I**) are zero, and the largest excitation wavelengths (with small oscillator strength) for them are at 970, 1084 and 994 nm. The computed results show that Li&B_44_^−^ (**I**) and Na&B_44_^−^ (**I**) have the similar electronic absorption spectra. Figure 8(k–o) present the electronic absorption spectra of metalloborospherenes MB_44_^−^ (**IV**), the calculated largest excitation wavelengths of Li&B_44_^−^ (**IV**), Na&B_44_^−^ (**IV**), Na@B_44_^−^ (**IV**), K&B_44_^−^ (**IV**) and K@B_44_^−^ (**IV**) are at 1348, 1340, 1392 1353 and 1617 nm, respectively. Figure 8(a–e) indicate that the largest excitation wavelengths of B_44_ (except for **V**) are in the near infrared region. One can observe several near infrared (NIR) absorption peaks of the B_44_ (**I**, **II**, **III**, and **IV**), whereas B_44_ (**V**) has only UV-Vis absorption peaks. Figure 8(f–o) indicate that the largest excitation wavelengths of metalloborospherenes MB_44_^−^ (M = Li, Na, and K) are in the near infrared region. One can observe several near infrared (NIR) absorption peaks of metalloborospherenes MB_44_^−^ (M = Li, Na, and K).

Figure 8(a) and (f–j) indicate that doping of metal atom in B_44_ (**I**) blueshifts the largest excitation wavelength since doping of metal atom in B_44_ (**I**) leads to the increase of energy gap (as shown in Tables 1, 2). However, Fig. 8(d) and 8(k–o) indicate that doping of metal atom in B_44_ (**IV**) redshifts the largest excitation wavelength since doping of metal atom in B_44_ (**IV**) leads to the decrease of energy gap (as shown in Tables 1, 2). It’s worth noting that the electronic absorption spectra of exohedral M&B_44_^−^ (**I**, M = Li, Na, and K) and endohedral M@B_44_^−^ (**I**, M = Na, and K) are similar to that of dianion B_44_^2−^ (**I**) (Figure S4). Similarly, the electronic absorption spectra of exohedral M&B_44_^−^ (**IV**, M = Li, Na, and K) and endohedral M@B_44_^−^ (**IV**, M = Na, and K) are similar to that of dianion B_44_^2−^ (**IV**) (Figure S4). It further suggests that B_44_ (**I**, **IV**) tend to get two electrons from the extra electron and the doped metal, respectively. The electronic absorption spectra may be used for the structural analysis in conjunction with other techniques. In addition, UV-Vis spectroscopy can be used to distinguish isomers, such as the five isomers of B_44_ with obvious different absorption peaks.

In a summary, the structures, stabilities, photoelectron spectra, infrared spectra, Raman spectra, and electronic absorption spectra of B_44_^−^ and metalloborospherenes MB_44_^0/−^ (M = Li, Na, and K) were studied at the level of density functional theory (DFT) and time-dependent density functional theory (TD-DFT) with 6–311 + G* basis set. The calculated results suggest that Li, Na and K atoms can form stable exohedral M&B_44_^0/−^ (M = Li, Na, and K), whereas only Na and K atoms can be stably encapsulated inside the B_44_^0/−^ cage. In addition, relative energies of these metalloborospherenes reveal that the Na and K atoms favor the exohedral configuration. More importantly, the addition of metal atom can modify the stability of B_44_ with different structures, which provides a possible route (doping of metal atoms) to produce stable boron clusters or metalloborospherenes. The calculated results suggest that B_44_ tends to get electrons from the doped metal. Metalloborospherenes MB_44_^−^ are characterized as charge-transfer complexes (M^2+^B_44_^2−^), where B_44_ tends to get two electrons from the extra electron and the doped metal, resulting in similar features with anionic B_44_^2−^. The calculated results show that B_44_^−^ and metalloborospherenes MB_44_^0/−^ (M = Li, Na, and K) have different and meaningful spectral features, insight into the spectral properties is important to understand them and find their potential applications. In addition, the calculated electronic absorption spectra indicate that B_44_ and metalloborospherenes MB_44_^−^ (M = Li, Na, and K) have obvious near-IR absorption peaks. These spectral features can be used as fingerprints to identify and distinguish the borospherenes B_44_^0/−^ and metalloborospherenes MB_44_^0/−^ (M = Li, Na, and K). The all-boron fullerenes and metalloborospherenes have provided an important clue for the development of new boron-based materials. In view of the remarkable structures and properties, it is possible that borospherenes and metalloborospherenes have potential applications in energy, environment, optoelectronic materials and pharmaceutical chemistry.

## Additional Information

**How to cite this article**: Li, S. *et al*. Structures, stabilities and spectral properties of borospherene B_44_ ^−^ and metalloborospherenes MB_44_^0/−^ (M = Li, Na, and K). *Sci. Rep.* **7**, 40081; doi: 10.1038/srep40081 (2017).

**Publisher's note:** Springer Nature remains neutral with regard to jurisdictional claims in published maps and institutional affiliations.

## Supplementary Material

Supplementary Information

## Figures and Tables

**Table 1:** The symmetries, energies (*E*), relative energies (neutral B_44_: the energy of B_44_ (IV) is set to be zero, anionic B_44_^−^: the energy of B_44_^−^ (I) is set to be zero), energy gaps (*E*_g_), dipole moments (μ) and states of borospherenes B_44_^0/−^ optimized at PBE0/6–311 + G* level.

	Isomer	Symmetry	*E*/hartree	relative energy/eV	*E*_g_/eV	μ/Debye	State
B_44_	**I**	C_2v_	−1091.8943	0.17	1.3524	2.0800	^1^A_1_
B_44_	**II**	C_1_	−1091.8956	0.14	1.8515	1.3011	^1^A
B_44_	**III**	C_1_	−1091.8955	0.14	2.2410	1.6519	^1^A
B_44_	**IV**	C_2_	−1091.9007	0.00	2.1205	0.9490	^1^A
B_44_	**V**	C_1_	−1091.8920	0.24	2.5946	1.7655	^1^A
B_44_^−^	**I**	C_2v_	−1092.0129	0.00	1.9769^a^	2.5928	^2^A_2_
			−1093.6775^c^	[0.00]	1.2716^b^		
			−1081.8145^d^	(0.00)			
B_44_^−^	**II**	C_1_	−1092.0068	0.17	2.1088^a^	2.5478	^2^A
			−1093.6719^c^	[0.15]	1.9092^b^		
			−1081.7928^d^	(0.59)			
B_44_^−^	**III**	C_1_	−1092.0068	0.17	1.9628^a^	2.5600	^2^A
			−1093.6719^c^	[0.15]	1.9078^b^		
			−1081.7928^d^	(0.59)			
B_44_^−^	**IV**	C_2_	−1092.0028	0.27	1.4938^a^	2.1723	^2^A
			−1093.6688^c^	[0.24]	1.9168^b^		
			−1081.7963^d^	(0.50)			
B_44_^−^	**V**	C_1_	−1092.0021	0.29	2.0737^a^	3.9325	^2^A
			−1093.6682^c^	[0.25]	2.2908^b^		
			−1081.7925^d^	(0.60)			

The superscripts a and b denote the alpha electron and beta electron, respectively. The superscripts c and d denote the energies (*E)* of B_44_^−^ at TPSSh/6–311 + G* and UCCSD(T)/3–21 G//PBE0/6–311 + G* levels. The square bracket and round bracket denote the relative energies of B_44_^−^ at TPSSh/6–311 + G* and UCCSD(T)/3–21 G//PBE0/6–311 + G* levels.

**Table 2:** The symmetries, energies (*E)*, relative energies (the round bracket denotes the relative energies of LiB_44_^0/−^ and the energy of Li&B_44_^−^ (I) is set to be zero; the square bracket denotes the relative energies of NaB_44_^0/−^ and the energy of Na&B_44_^−^ (I) is set to be zero; the curly bracket denotes the relative energies of KB_44_^0/−^ and the energy of K&B_44_^−^ (I) is set to be zero), energy gaps (*E*_g_), dipole moments (μ) and states of metalloborospherenes MB_44_^0/−^ (M = Li, Na, and K) optimized at PBE0/6–311 + G* level.

MB_44_^0/−^	B_44_ structure	Symmetry	*E*/hartree	relative energy/eV	*E*_g_/eV	μ/Debye	State
Li&B_44_	**I**	C_s_	−1099.4814	(3.17)	2.0305^a^, 1.6228^b^	2.6775	^2^A”
Na&B_44_	**I**	C_s_	−1254.1542	[3.01]	2.0193^a^, 1.5917^b^	5.9058	^2^A”
Na@B_44_	**I**	C_2v_	−1254.1450	[3.26]	1.8545^a^, 1.3614^b^	2.4171	^2^A_2_
K&B_44_	**I**	C_s_	−1691.7302	{2.87}	2.0196^a^, 1.5711^b^	8.9039	^2^A”
K@B_44_	**I**	C_2v_	−1691.7140	{3.31}	1.8537^a^, 1.3461^b^	2.4406	^2^A_2_
Li&B_44_^−^	**I**	C_s_	−1099.5978	(0.00)	1.9491	4.3131	^1^A’
Na&B_44_^−^	**I**	C_s_	−1254.2647	[0.00]	1.9357	7.7502	^1^A’
Na@B_44_^−^	**I**	C_2v_	−1254.2640	[0.02]	1.8848	3.0687	^1^A_1_
K&B_44_^−^	**I**	C_s_	−1691.8357	{0.00}	1.9491	11.1740	^1^A’
K@B_44_^−^	**I**	C_2v_	−1691.8325	{0.09}	1.8832	3.0501	^1^A_1_
Li&B_44_	**IV**	C_1_	−1099.4726	(3.41)	1.6793^a^, 2.0169^b^	1.7322	^2^A
Na&B_44_	**IV**	C_1_	−1254.1494	[3.14]	1.6611^a^, 2.0449^b^	4.9882	^2^A
Na@B_44_	**IV**	C_2_	−1254.1427	[3.32]	1.6587^a^, 1.7887^b^	1.0043	^2^A
K&B_44_	**IV**	C_1_	−1691.7234	{3.06}	1.6426^a^, 2.0335^b^	8.1784	^2^A
K@B_44_	**IV**	C_2_	−1691.7009	{3.67}	1.4884^a^, 1.8346^b^	1.8479	^2^A
Li&B_44_^−^	**IV**	C_1_	−1099.5813	(0.45)	1.7096	1.7304	^1^A
Na&B_44_^−^	**IV**	C_1_	−1254.2518	[0.35]	1.7009	4.2752	^1^A
Na@B_44_^−^	**IV**	C_2_	−1254.2569	[0.21]	1.6819	1.9305	^1^A
K&B_44_^−^	**IV**	C_1_	−1691.8202	{0.42}	1.6824	7.7875	^1^A
K@B_44_^−^	**IV**	C_2_	−1691.8116	{0.66}	1.5668	2.9593	^1^A

The superscripts a and b denote the alpha electron and beta electron, respectively.
