# Supplementary material for: Structures, stabilities and spectral properties of borospherene B44− and metalloborospherenes MB440/− (M = Li, Na, and K)
Source: Sci Rep. 2017 Jan 10;7:40081. doi: 10.1038/srep40081 (PMC5223222; doi:10.1038/srep40081)
Supplement: Supplementary Information [file srep40081-s1.doc]

**Structures, stabilities and spectral properties of borospherene B_44_**^−^ **and metalloborospherenes MB_44_^0/^**^−^ **(M=Li, Na, and K)**

Shixiong Li ^1, 2^, Zhengping Zhang ^1,*^, Zhengwen Long ^3^ & Shuijie Qin ^4^

1 College of Big Data and Information Engineering, Guizhou University, Guiyang 550025, China

2 School of Physics and Electronic Science, Guizhou Education University, Guiyang 550018, China

3 College of Science, Guizhou University, Guiyang 550025, China

4 Key Lab of Photoelectron Technology and Application, Guizhou University, Guiyang 550025, China

email: [zpzhang@gzu.edu.cn](mailto:zpzhang@gzu.edu.cn)

email: [leesxoptics@163.com](mailto:leesxoptics@163.com)

**Figure S1**. Optimized Structures of metalloborospherenes MB_44_^0/−^ (M=Li, Na, and K) at the PBE0/6-311+G* level.


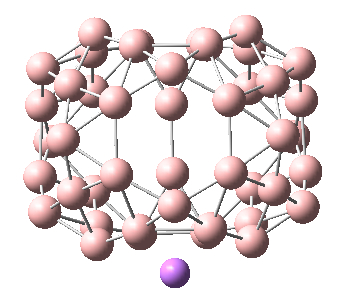

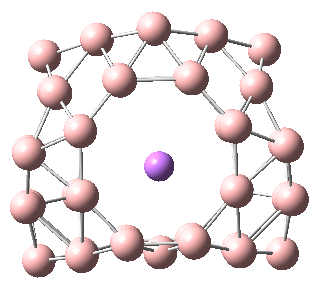


Top Side

(a) C_s_ Li&B_44_^0/−^ (**I**)


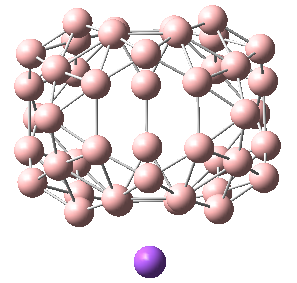

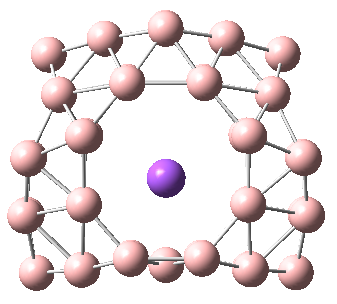


Top Side

(b) C_s_ Na&B_44_^0/−^ (**I**)


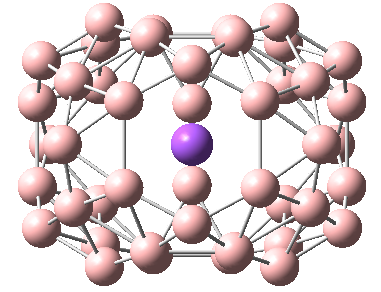

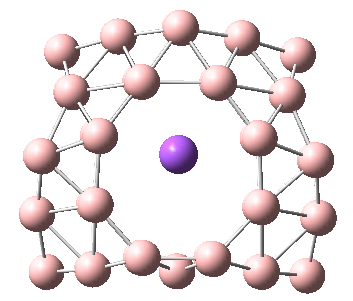


Top Side

(c) C_2v_ Na@B_44_^0/−^ (**I**)


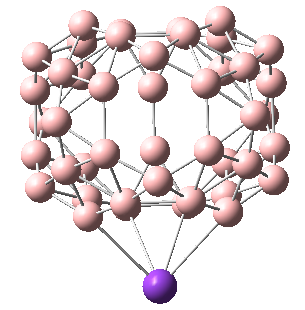

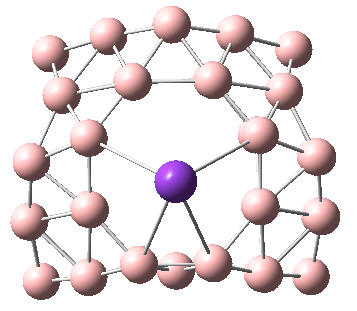


Top Side

(d) C_s_ K&B_44_^0/−^ (**I**)


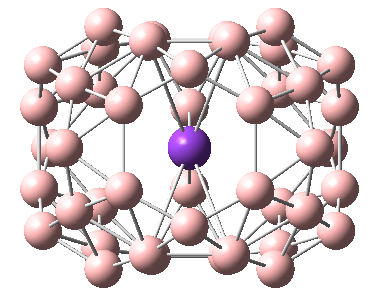

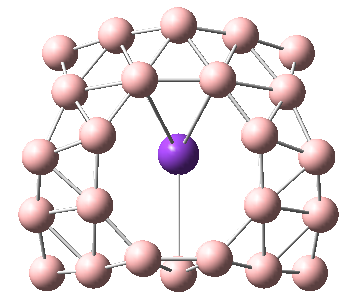


Top Side

(e) C_2v_ K@B_44_^0/−^ (**I**)


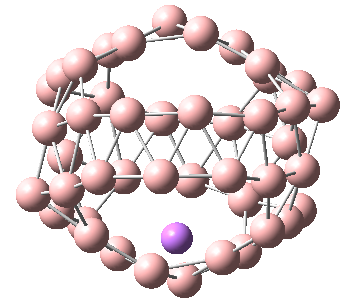

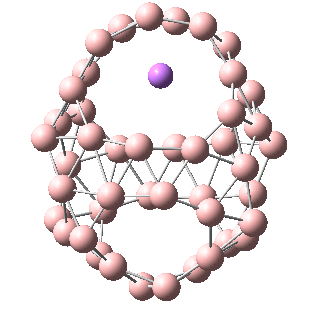


Top Side

(f) C_1_ Li&B_44_^0/−^ (**IV**)


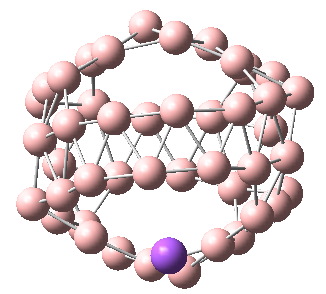

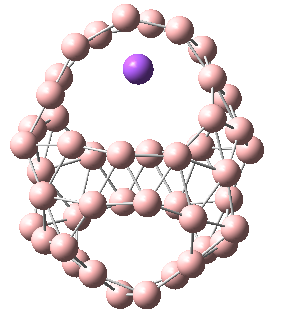


Top Side

(g) C_1_ Na&B_44_^0/−^ (**IV**)


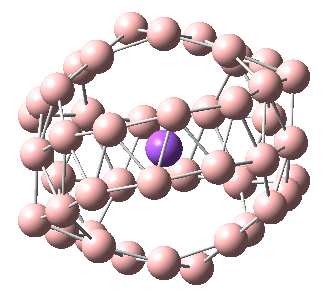

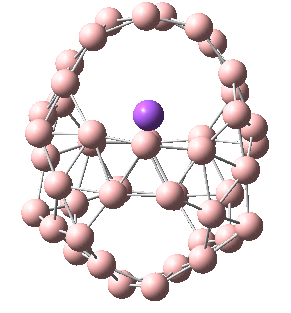


Top Side

(h) C_2_ Na@B_44_^0/−^ (**IV**)


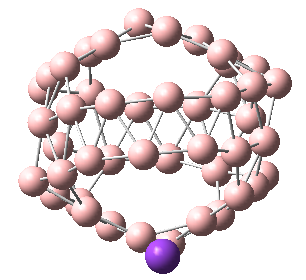

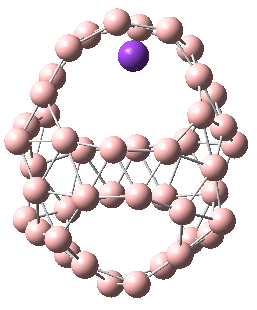


Top Side

(i) C_1_ K&B_44_^0/−^ (**IV**)


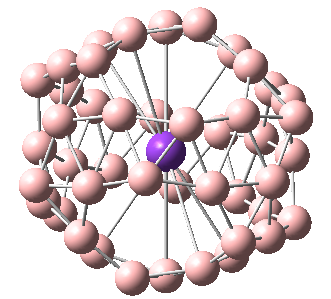

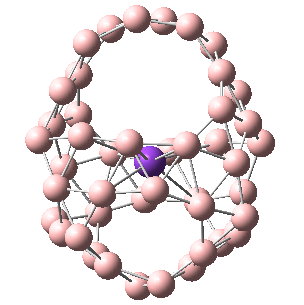


Top Side

(j) C_2_ K@B_44_^0/−^ (**IV**)

**Figure S2**. Infrared spectra of B_44_^2−^  at the PBE0/6-311+G* level. (a) B_44_^2−^(**I**), (b) B_44_^2−^ (**IV**).

**Figure S3**. Raman spectra of B_44_^2−^  at the PBE0/6-311+G* level. (a) B_44_^2−^(**I**), (b) B_44_^2−^ (**IV**).

**Figure S4**. Electronic absorption spectra of B_44_^2−^  at the PBE0/6-311+G* level. (a) B_44_^2−^(**I**), (b) B_44_^2−^ (**IV**).

**Table S1**. The symmetries, energies (*E*), relative energies (the energy of B_44_^2−^ (**I**) is set to be zero), energy gaps (*E*_g_), dipole moments (μ) and states of borospherenes B_44_^2−^ optimized at PBE0/6-311+G* level.

| B_44_^2−^ | B_44_ structure | Symmetry | *E*/hartree | relative energy/eV | *E*_g_/eV | μ/ Debye | State |
| --- | --- | --- | --- | --- | --- | --- | --- |
| B_44_^2−^ | **I** | C_2v_ | -1092.0230 | 0.00 | 1.99 | 3.2096 | ^1^ A_1_ |
| B_44_^2−^ | **IV** | C_2_ | -1091.9985 | 0.67 | 1.52 | 3.5925 | ^1^ A |

**Table S2**. Optimized coordinates of borospherenes B_44_^0/−^ at the PBE0/6-311+G* level.

C_2v_ B_44_ (**I**)

B -3.14809200 1.74335800 0.07531300

B 0.00000000 1.64581100 3.02678700

B -2.51965600 1.33838700 1.55442500

B 3.14809200 -1.74335800 0.07531300

B 3.01648200 0.00000000 -2.60969500

B -0.79671400 2.29398300 -2.19996900

B 0.86304000 -2.25451000 1.80207600

B -1.94089600 -2.15476200 -1.02778600

B -3.14809200 -1.74335800 0.07531300

B 0.00000000 -1.64581100 3.02678700

B -2.51965600 -1.33838700 1.55442500

B 1.83420600 2.53954000 0.55495800

B 2.51965600 1.33838700 1.55442500

B 2.51965600 -1.33838700 1.55442500

B 3.23369800 -0.86803500 -1.25871100

B -3.23369800 0.86803500 -1.25871100

B -0.86304000 -2.25451000 1.80207600

B 3.23369800 0.86803500 -1.25871100

B 2.72413000 0.00000000 2.44151000

B -1.83420600 2.53954000 0.55495800

B -0.86304000 2.25451000 1.80207600

B 0.86304000 2.25451000 1.80207600

B 1.94089600 -2.15476200 -1.02778600

B -1.94089600 2.15476200 -1.02778600

B 1.94089600 2.15476200 -1.02778600

B 1.42078000 -0.87863000 2.82340100

B 1.42078000 0.87863000 2.82340100

B 3.14809200 1.74335800 0.07531300

B 1.92014400 1.19797900 -2.46951100

B -0.79671400 -2.29398300 -2.19996900

B -3.23369800 -0.86803500 -1.25871100

B -1.42078000 -0.87863000 2.82340100

B -1.42078000 0.87863000 2.82340100

B -1.92014400 1.19797900 -2.46951100

B 1.83420600 -2.53954000 0.55495800

B -2.72413000 0.00000000 2.44151000

B 1.92014400 -1.19797900 -2.46951100

B -3.01648200 0.00000000 -2.60969500

B -1.83420600 -2.53954000 0.55495800

B -1.92014400 -1.19797900 -2.46951100

B 0.00000000 0.85224100 -2.56699200

B 0.79671400 2.29398300 -2.19996900

B 0.00000000 -0.85224100 -2.56699200

B 0.79671400 -2.29398300 -2.19996900

C_2v_ B_44_^−^ (**I**)

B -3.15078700 1.75966700 0.07036800

B 0.00000000 1.67276500 3.04115100

B -2.48909300 1.32000600 1.53917200

B 3.15078700 -1.75966700 0.07036800

B 2.99696400 0.00000000 -2.59694000

B -0.80466600 2.31847100 -2.22246200

B 0.87442900 -2.25419500 1.80163300

B -1.91817800 -2.14440500 -1.02289900

B -3.15078700 -1.75966700 0.07036800

B 0.00000000 -1.67276500 3.04115100

B -2.48909300 -1.32000600 1.53917200

B 1.84531600 2.56544000 0.55765400

B 2.48909300 1.32000600 1.53917200

B 2.48909300 -1.32000600 1.53917200

B 3.22466000 -0.87566700 -1.25618200

B -3.22466000 0.87566700 -1.25618200

B -0.87442900 -2.25419500 1.80163300

B 3.22466000 0.87566700 -1.25618200

B 2.71341300 0.00000000 2.45146000

B -1.84531600 2.56544000 0.55765400

B -0.87442900 2.25419500 1.80163300

B 0.87442900 2.25419500 1.80163300

B 1.91817800 -2.14440500 -1.02289900

B -1.91817800 2.14440500 -1.02289900

B 1.91817800 2.14440500 -1.02289900

B 1.41274900 -0.89458900 2.82200900

B 1.41274900 0.89458900 2.82200900

B 3.15078700 1.75966700 0.07036800

B 1.92352400 1.23301800 -2.49160500

B -0.80466600 -2.31847100 -2.22246200

B -3.22466000 -0.87566700 -1.25618200

B -1.41274900 -0.89458900 2.82200900

B -1.41274900 0.89458900 2.82200900

B -1.92352400 1.23301800 -2.49160500

B 1.84531600 -2.56544000 0.55765400

B -2.71341300 0.00000000 2.45146000

B 1.92352400 -1.23301800 -2.49160500

B -2.99696400 0.00000000 -2.59694000

B -1.84531600 -2.56544000 0.55765400

B -1.92352400 -1.23301800 -2.49160500

B 0.00000000 0.85551300 -2.49104600

B 0.80466600 2.31847100 -2.22246200

B 0.00000000 -0.85551300 -2.49104600

B 0.80466600 -2.31847100 -2.22246200

C_1_ B_44_ (**II**)

B -1.79823300 -2.02204700 -1.68673400

B -3.14514000 -1.05720200 -1.51106000

B -1.97133400 1.60650600 -2.04496000

B -0.80440000 1.06241800 -2.97558800

B 0.97589400 -1.66081700 -2.14250900

B 1.72993500 0.01530000 -2.19696900

B -0.22521200 2.25232100 -1.91389400

B -3.32098100 -0.64279100 0.12158500

B -1.50168800 -2.89089300 -0.08966600

B 0.08928000 -0.35359200 -2.82879700

B 0.75500200 1.18320600 -2.74259700

B -3.10958000 0.52970900 -1.28446500

B -2.89482300 -2.16638200 -0.41276500

B -0.54667600 -1.83125400 -2.68825500

B 2.42592700 -1.38477400 -1.46927100

B -0.25275400 -2.73848400 -1.37026400

B -1.44132000 2.77752300 -0.79933000

B -2.92584000 2.07499000 -0.82427100

B -3.22703700 1.05437600 0.42199800

B 0.53366600 -1.69244600 2.37506700

B -0.38721500 -0.51924600 3.11332600

B 1.95601800 2.02811800 1.67088800

B 3.11312500 1.00679100 1.01868800

B 2.08154800 -1.68402000 1.95592500

B 3.73279800 1.54582100 -0.51013800

B 1.08524900 2.80721200 0.47421800

B -1.94289100 -0.39593000 2.43782600

B -0.62330300 -2.77829000 1.41720400

B 4.23493400 0.16516400 0.10566500

B 2.66935600 2.45385300 0.25179900

B -0.72808000 1.00167300 2.73692800

B -1.13048300 -1.82905400 2.60386200

B 3.66450000 -1.28303600 -0.36862600

B 1.11975300 -2.71324800 1.09758900

B -0.59326700 2.97578000 0.73573500

B -1.22565300 2.41471900 2.12164800

B 0.39438700 2.15715100 2.00584900

B -2.30417400 1.20995600 1.94031900

B 0.09163100 3.29127200 -0.70979500

B 3.15388400 0.12928300 -1.26566800

B 0.02509200 -3.41882100 0.08078200

B -3.28873600 -0.04537100 1.62040600

B 2.31411400 -1.85108600 0.30231500

B 3.24272500 -0.78435900 1.22600200

C_1_ B_44_^−^ (**II**)

B -1.74660000 -2.07988300 -1.64190200

B -3.13519400 -1.17790400 -1.45049200

B -2.05142600 1.51270400 -2.00605600

B -0.89245000 1.00813500 -2.97515800

B 0.98585900 -1.61308300 -2.13354500

B 1.70411800 0.06541500 -2.19194200

B -0.37773900 2.22928500 -1.92358300

B -3.29383400 -0.75917300 0.18225600

B -1.39818100 -2.96650800 -0.05435800

B 0.05765700 -0.37120100 -2.87242500

B 0.66061500 1.18052500 -2.69484600

B -3.16221900 0.41475500 -1.23810800

B -2.81167200 -2.27149700 -0.34919400

B -0.53308600 -1.86355100 -2.68395400

B 2.40648500 -1.30530100 -1.41398900

B -0.17719900 -2.75620500 -1.36780600

B -1.58875800 2.74391400 -0.79098400

B -3.03780600 1.97561200 -0.79423400

B -3.25712900 0.94497600 0.46109100

B 0.65466600 -1.65348700 2.34928200

B -0.29240000 -0.49529300 3.08760500

B 1.88135500 2.08776000 1.60590900

B 3.02054100 1.06737200 0.94025000

B 2.22726800 -1.63925800 1.95604600

B 3.70096400 1.65513800 -0.55408200

B 0.98699300 2.86200100 0.40842100

B -1.87350200 -0.43375100 2.45386000

B -0.48086000 -2.77056900 1.42151700

B 4.25269000 0.29024500 0.06841800

B 2.60236200 2.53565400 0.20044400

B -0.71945600 1.00991800 2.72262200

B -1.00650000 -1.83322200 2.60553700

B 3.75792400 -1.19745000 -0.41718400

B 1.25315000 -2.67212600 1.07681600

B -0.70016500 2.99166900 0.72587700

B -1.29315000 2.40490200 2.11927200

B 0.33195800 2.20821200 1.96130500

B -2.32140400 1.15298800 1.96765900

B -0.06755000 3.30451400 -0.74142600

B 3.13767800 0.21560400 -1.29185300

B 0.14630600 -3.43401600 0.08721700

B -3.26108500 -0.14383300 1.67417000

B 2.40477400 -1.76956500 0.28601100

B 3.30600000 -0.65441700 1.22553200

C_1_ B_44_ (**III**)

B -1.62413500 1.78074900 2.01773900

B -3.06177700 0.98162600 1.71224400

B -2.08125400 -1.79928100 1.76863100

B -0.89441200 -1.52959700 2.79863400

B 1.10567200 1.15484500 2.45991000

B 1.76739300 -0.58391600 2.27104900

B -0.40404000 -2.54124600 1.53380000

B -3.27113600 0.86542700 0.03680500

B -1.24945100 2.84991800 0.57730300

B 0.08821700 -0.19322100 2.91986900

B 0.65347700 -1.72469900 2.54019800

B -3.16150700 -0.54153000 1.22371400

B -2.70810600 2.22892700 0.80770200

B -0.41386100 1.34291900 2.98942400

B 2.59697900 0.81186200 1.96601500

B -0.01630700 2.37256600 1.78211600

B -1.63789400 -2.77369600 0.34067400

B -3.07193000 -1.99517700 0.50288600

B -3.27903600 -0.75406500 -0.54545700

B 0.66744200 2.06005100 -2.22274700

B -0.39496300 1.05959600 -3.04745000

B 1.80072100 -1.80009800 -1.98268300

B 2.98928400 -0.95570000 -1.17668200

B 2.20929900 1.94663300 -1.83649000

B 3.64980800 -1.79761800 0.18536600

B 0.89941300 -2.72329800 -0.91406200

B -1.91533500 0.91861500 -2.32686900

B -0.42406200 3.00933700 -0.96394400

B 4.18455000 -0.34299500 -0.13435000

B 2.52209500 -2.50754400 -0.68466600

B -0.80687900 -0.48440500 -2.88910500

B -1.01715600 2.30336400 -2.28237200

B 3.51658700 1.03673600 0.56513500

B 1.30121900 2.84555000 -0.81529500

B -0.78981100 -2.76783700 -1.21396800

B -1.38403600 -1.94105100 -2.47877200

B 0.24206400 -1.79291200 -2.32043300

B -2.37933300 -0.71289100 -2.07729200

B -0.13480200 -3.35363500 0.15771300

B 3.06550500 -0.56054800 1.23254400

B 0.32239200 3.24931500 0.44942600

B -3.27068000 0.53901200 -1.54250300

B 2.61298200 2.06050700 -0.28409200

B 3.19680300 0.75940300 -1.09966300

C_1_ B_44_^−^ (**III**)

B -1.74635500 -2.08045200 -1.64107400

B -3.13541300 -1.17940200 -1.44919600

B -2.05304200 1.51139600 -2.00574800

B -0.89415200 1.00755400 -2.97529700

B 0.98523500 -1.61289400 -2.13392300

B 1.70241300 0.06522500 -2.19139400

B -0.37867200 2.22830600 -1.92339800

B -3.29378300 -0.76015900 0.18341900

B -1.39669500 -2.96615300 -0.05324800

B 0.05555100 -0.37208800 -2.87334300

B 0.65882400 1.17971200 -2.69568300

B -3.16342400 0.41327500 -1.23684000

B -2.81090600 -2.27247900 -0.34773200

B -0.53368000 -1.86487300 -2.68436800

B 2.40497500 -1.30397200 -1.41335200

B -0.17667600 -2.75638100 -1.36762000

B -1.58982800 2.74283000 -0.79115300

B -3.03865300 1.97412700 -0.79348900

B -3.25742800 0.94389800 0.46223000

B 0.65660100 -1.65296400 2.34966700

B -0.29080400 -0.49461800 3.08750900

B 1.88124900 2.08988300 1.60473800

B 3.01979400 1.06885300 0.93898600

B 2.22877100 -1.63768400 1.95595400

B 3.69978300 1.65594300 -0.55609700

B 0.98615300 2.86333200 0.40731000

B -1.87221200 -0.43375400 2.45481800

B -0.47873500 -2.77044700 1.42229400

B 4.25202600 0.29168100 0.06762800

B 2.60135900 2.53666200 0.19859500

B -0.71844600 1.01029400 2.72236300

B -1.00454300 -1.83289900 2.60611200

B 3.75860900 -1.19681900 -0.41810300

B 1.25507600 -2.67176600 1.07718400

B -0.70090800 2.99115300 0.72537300

B -1.29343100 2.40498600 2.11920600

B 0.33176900 2.20952100 1.96068400

B -2.32129600 1.15271700 1.96849300

B -0.06920800 3.30483900 -0.74229300

B 3.13736400 0.21584600 -1.29317400

B 0.14781900 -3.43324300 0.08759800

B -3.26005500 -0.14492800 1.67536500

B 2.40772600 -1.77103900 0.28629000

B 3.30724900 -0.65302000 1.22470800

C_2_ B_44_ (**IV**)

B 1.23892600 0.61739300 -3.10053100

B -0.33767400 0.71403700 -3.64835500

B -1.54438200 2.31349800 -1.46123100

B -0.16710100 2.94193100 -0.98684100

B 2.81863600 1.43045700 -0.88402900

B 1.97562900 1.97120600 0.64289800

B -1.09270900 2.45995900 0.34757100

B -1.23892600 -0.61739300 -3.10053100

B 1.60599200 -1.17462100 -2.78247400

B 1.37497500 2.34918400 -1.03313800

B 0.57020700 2.72805200 0.41519800

B -1.60599200 1.17462100 -2.78247400

B 0.33767400 -0.71403700 -3.64835500

B 2.13490200 1.70493000 -2.32604300

B 3.35086900 1.05599100 0.58516600

B 2.60304400 0.15820500 -2.14912900

B -2.58675200 1.68956900 -0.15735600

B -2.84496300 1.41118500 -1.76192800

B -2.60304400 -0.15820500 -2.14912900

B 1.09270900 -2.45995900 0.34757100

B -0.57020700 -2.72805200 0.41519800

B -1.90328700 -0.70605300 3.42152700

B 1.19963100 -0.69046700 3.89748100

B 2.19320400 -1.91767500 1.41104300

B 1.90328700 0.70605300 3.42152700

B -2.08457200 0.76087100 2.52376900

B -1.37497500 -2.34918400 -1.03313800

B 1.54438200 -2.31349800 -1.46123100

B 2.08457200 -0.76087100 2.52376900

B -1.19963100 0.69046700 3.89748100

B -1.97562900 -1.97120600 0.64289800

B 0.16710100 -2.94193100 -0.98684100

B 2.84496300 -0.45740800 1.01937500

B 2.58675200 -1.68956900 -0.15735600

B -2.84496300 0.45740800 1.01937500

B -3.35086900 -1.05599100 0.58516600

B -2.64572800 -0.99222300 2.01735200

B -2.81863600 -1.43045700 -0.88402900

B -2.19320400 1.91767500 1.41104300

B 2.64572800 0.99222300 2.01735200

B 2.84496300 -1.41118500 -1.76192800

B -2.13490200 -1.70493000 -2.32604300

B -0.38070100 -0.76971200 4.00967700

B 0.38070100 0.76971200 4.00967700

C_2_ B_44_^−^ (**IV**)

B 1.22084700 0.64080500 -3.09726500

B -0.35275600 0.70672600 -3.66465400

B -1.58016100 2.27591500 -1.45029900

B -0.21668200 2.94498900 -0.98368200

B 2.77604900 1.47284100 -0.87931000

B 1.95309200 1.96256400 0.62949800

B -1.14587500 2.42647800 0.34025800

B -1.22084700 -0.64080500 -3.09726500

B 1.62322000 -1.14580100 -2.78360700

B 1.33041900 2.38924500 -1.05349000

B 0.51823500 2.67923700 0.41845500

B -1.62322000 1.14580100 -2.78360700

B 0.35275600 -0.70672600 -3.66465400

B 2.10825100 1.74712500 -2.33993400

B 3.34233200 1.09290600 0.56829800

B 2.59653200 0.20856500 -2.15971100

B -2.63919700 1.64855700 -0.15269000

B -2.86759200 1.35690100 -1.76433000

B -2.59653200 -0.20856500 -2.15971100

B 1.14587500 -2.42647800 0.34025800

B -0.51823500 -2.67923700 0.41845500

B -1.88733700 -0.72188800 3.41221900

B 1.18676400 -0.68713700 3.86779200

B 2.24084300 -1.87878800 1.41477900

B 1.88733700 0.72188800 3.41221900

B -2.18315100 0.71423400 2.54337800

B -1.33041900 -2.38924500 -1.05349000

B 1.58016100 -2.27591500 -1.45029900

B 2.18315100 -0.71423400 2.54337800

B -1.18676400 0.68713700 3.86779200

B -1.95309200 -1.96256400 0.62949800

B 0.21668200 -2.94498900 -0.98368200

B 2.95264200 -0.44135700 1.04323700

B 2.63919700 -1.64855700 -0.15269000

B -2.95264200 0.44135700 1.04323700

B -3.34233200 -1.09290600 0.56829800

B -2.63919700 -0.98015900 2.02139800

B -2.77604900 -1.47284100 -0.87931000

B -2.24084300 1.87878800 1.41477900

B 2.63919700 0.98015900 2.02139800

B 2.86759200 -1.35690100 -1.76433000

B -2.10825100 -1.74712500 -2.33993400

B -0.37989800 -0.75544000 4.06965700

B 0.37989800 0.75544000 4.06965700

C_1_ B_44_ (**V**)

B -2.91390200 -1.42698600 0.95703500

B -1.86699300 -2.63060000 1.43052000

B 0.28469700 -1.02064800 2.69225300

B -0.68371300 0.18116500 3.11627800

B -2.95716300 1.40298500 1.01418100

B -1.52206200 2.35635900 1.51891900

B 0.78020600 0.77740200 2.49226800

B -0.80914500 -3.08664400 0.19834400

B -2.98761500 -1.45711200 -0.90368100

B -2.00214000 0.78371500 2.31354500

B -0.61386800 1.68703500 2.65132600

B -0.33743200 -2.43121700 1.84748200

B -2.41707500 -2.68839900 -0.05246700

B -3.25473400 -0.04111000 1.69866700

B -2.42379500 2.72325600 0.21809900

B -3.43871100 -0.03629900 0.08172400

B 1.81402900 -0.54073100 1.96358800

B 1.24457700 -2.09881300 1.99971100

B 0.87472300 -2.82352200 0.59258100

B -0.73150100 0.30398300 -2.78056500

B 0.66925500 -0.62861400 -2.82317400

B 2.70037500 1.55367700 -1.07024400

B 1.56640700 2.65737300 -1.30566700

B -0.93376700 1.89716500 -2.56575900

B 0.71093600 3.29276600 -0.09727300

B 3.40744500 1.62916400 0.71830000

B 0.44847700 -2.07698600 -1.96211900

B -2.12014400 -0.78271600 -2.27042800

B -0.07488000 2.70523400 -1.46414000

B 2.00117100 2.30944500 0.31116100

B 1.99202100 -0.96113500 -1.94802300

B -0.74639600 -1.37400500 -2.82542900

B -1.74886300 2.41500800 -1.25251800

B -2.21648200 0.98695600 -2.13848100

B 3.20188200 -0.06839900 1.12808700

B 3.16180800 -1.67336100 -0.97853200

B 4.49813400 0.58997900 0.15199700

B 1.72518500 -2.43182700 -0.90408100

B 2.15297100 1.10935800 1.73939200

B -0.86307500 3.15280900 0.11725600

B -3.35926300 -0.01980900 -1.55029100

B 0.35619000 -3.29418800 -0.88523600

B 4.21644900 -0.91285800 -0.03306500

B 3.21578100 -0.00885500 -1.14154000

C_1_ B_44_^−^ (**V**)

B 2.91283200 -1.26323800 0.65224700

B 3.37502500 0.33364200 0.72466000

B 1.09309500 1.49734300 2.22542100

B 0.75152000 0.14555900 3.01153500

B 0.67162000 -2.79537400 1.45437000

B -0.81488400 -2.02246000 2.09943200

B -0.66975600 0.90660800 2.47420300

B 2.91892300 1.19985200 -0.64899500

B 2.63964600 -1.68012800 -1.14223900

B 0.86632100 -1.42015900 2.48139800

B -0.58386600 -0.69248400 2.95555800

B 2.42551800 1.56843500 1.08087500

B 3.46237500 -0.38458600 -0.68306900

B 2.12613500 -2.13355600 1.75247000

B -0.83701200 -3.21966900 1.00122100

B 1.93164300 -2.61094100 0.20888600

B -0.29069700 2.35321600 1.55257100

B 1.29111200 2.72249500 1.21387900

B 1.82673200 2.52300400 -0.30774000

B -0.39491100 -1.13915700 -2.62985900

B -0.44488900 0.50096700 -3.00557800

B -3.02942600 1.35884400 -0.87371900

B -3.31862800 -0.20873600 -0.73768000

B -1.52272700 -2.11143300 -1.99111600

B -3.12557400 -1.00888000 0.64733600

B -3.16410900 2.25786500 0.82279400

B 1.00181000 1.26353500 -2.54352600

B 1.35750500 -1.59975000 -2.33595800

B -2.45767300 -1.61541400 -0.77289300

B -2.99004200 0.65765900 0.69953800

B -0.76612600 1.94085400 -2.33498000

B 0.95491400 -0.26623500 -3.11344900

B -1.23885800 -2.79476100 -0.53866100

B 0.01272600 -2.59555800 -1.73732200

B -1.60783900 3.07542900 0.78390700

B -0.67825400 3.47617200 -1.66366900

B -3.04842400 3.59505700 -0.06531700

B 0.75790600 2.71404300 -1.69979800

B -1.85040300 1.70775100 1.74912100

B -2.08363300 -2.18552200 0.91750900

B 1.57601500 -2.88097800 -1.36294900

B 2.22924100 2.05175400 -1.82109900

B -1.71412200 4.12723900 -0.62077500

B -2.07722200 2.60096700 -1.38623400

**Table S3**. Optimized coordinates of metalloborospherenes MB_44_^−^ (M=Li, Na, and K) at the PBE0/6-311+G* level.

C_s_ Li&B_44_^−^ (**I**)

B 0.05482300 1.71830200 3.18253500

B 3.03569700 1.68228200 0.00000000

B 1.52293500 1.27178000 2.46090700

B 0.08166100 -1.82295000 -3.15065700

B -2.57719100 -0.05811200 -2.98390000

B -2.24730100 2.26677700 0.82240800

B 1.81572300 -2.29001700 -0.88463300

B -1.00379100 -2.18231600 1.89555400

B 0.08166100 -1.82295000 3.15065700

B 3.06222000 -1.72355100 0.00000000

B 1.53603400 -1.34892800 2.46416500

B 0.57416000 2.56330700 -1.92745300

B 1.52293500 1.27178000 -2.46090700

B 1.53603400 -1.34892800 -2.46416500

B -1.23771700 -0.93628300 -3.20960900

B -1.25912700 0.82484400 3.25303000

B 1.81572300 -2.29001700 0.88463300

B -1.25912700 0.82484400 -3.25303000

B 2.45225000 -0.02514400 -2.69577600

B 0.57416000 2.56330700 1.92745300

B 1.78200500 2.23154600 0.89069300

B 1.78200500 2.23154600 -0.89069300

B -1.00379100 -2.18231600 -1.89555400

B -1.01213000 2.09828800 1.90443500

B -1.01213000 2.09828800 -1.90443500

B 2.81310300 -0.93255700 -1.39560200

B 2.81689100 0.88295800 -1.40606800

B 0.05482300 1.71830200 -3.18253500

B -2.49890100 1.20352700 -1.94854800

B -2.23274300 -2.39186000 0.81217600

B -1.23771700 -0.93628300 3.20960900

B 2.81310300 -0.93255700 1.39560200

B 2.81689100 0.88295800 1.40606800

B -2.49890100 1.20352700 1.94854800

B 0.57608500 -2.63247800 -1.85362700

B 2.45225000 -0.02514400 2.69577600

B -2.49949900 -1.31883100 -1.92575300

B -2.57719100 -0.05811200 2.98390000

B 0.57608500 -2.63247800 1.85362700

B -2.49949900 -1.31883100 1.92575300

B -2.40332300 0.79768800 0.00000000

B -2.24730100 2.26677700 -0.82240800

B -2.40892700 -0.91809900 0.00000000

B -2.23274300 -2.39186000 -0.81217600

Li -0.33367600 3.19661500 0.00000000

C_s_ Na&B_44_^−^ (**I**)

B -0.49863800 1.49109100 3.17769900

B 2.29841000 2.53074400 0.00000000

B 1.03357800 1.60454400 2.46098200

B 0.79748100 -1.80395400 -3.14964900

B -2.31790600 -1.11322700 -2.98582900

B -2.85635800 1.17420900 0.82406700

B 2.58765400 -1.62005000 -0.88559500

B -0.08493100 -2.52738000 1.89240800

B 0.79748100 -1.80395400 3.14964900

B 3.54650500 -0.64363500 0.00000000

B 1.98366500 -0.83909700 2.46107700

B -0.31917400 2.46015100 -1.91649700

B 1.03357800 1.60454400 -2.46098200

B 1.98366500 -0.83909700 -2.46107700

B -0.75206000 -1.44804500 -3.20941400

B -1.40979600 0.18985400 3.24973200

B 2.58765400 -1.62005000 0.88559500

B -1.40979600 0.18985400 -3.24973200

B 2.36409700 0.72413100 -2.69484800

B -0.31917400 2.46015100 1.91649700

B 0.93404700 2.59486200 0.89287800

B 0.93404700 2.59486200 -0.89287800

B -0.08493100 -2.52738000 -1.89240800

B -1.62955300 1.45365200 1.89542300

B -1.62955300 1.45365200 -1.89542300

B 3.02632800 0.00901600 -1.39294800

B 2.38040100 1.70736700 -1.40676200

B -0.49863800 1.49109100 -3.17769900

B -2.70650400 0.09081800 -1.95006900

B -1.15938700 -3.16594800 0.81220400

B -0.75206000 -1.44804500 3.20941400

B 3.02632800 0.00901600 1.39294800

B 2.38040100 1.70736700 1.40676200

B -2.70650400 0.09081800 1.95006900

B 1.55162100 -2.38306200 -1.85408300

B 2.36409700 0.72413100 2.69484800

B -1.79148500 -2.25717200 -1.92377400

B -2.31790600 -1.11322700 2.98582900

B 1.55162100 -2.38306200 1.85408300

B -1.79148500 -2.25717200 1.92377400

B -2.46251500 -0.24594000 0.00000000

B -2.85635800 1.17420900 -0.82406700

B -1.84606000 -1.84874000 0.00000000

B -1.15938700 -3.16594800 -0.81220400

Na -1.72840900 3.42002200 0.00000000

C_2v_ Na@B_44_^−^ (**I**)

B -3.15106100 1.75332400 0.06328500

B 0.00000000 1.67426300 3.05880200

B -2.47971200 1.31543300 1.54417200

B 3.15106100 -1.75332400 0.06328500

B 3.00151700 0.00000000 -2.59934800

B -0.81258400 2.30918600 -2.24474700

B 0.88420100 -2.24223000 1.80733500

B -1.91262300 -2.12790600 -1.03586900

B -3.15106100 -1.75332400 0.06328500

B 0.00000000 -1.67426300 3.05880200

B -2.47971200 -1.31543300 1.54417200

B 1.83694800 2.55615200 0.54604400

B 2.47971200 1.31543300 1.54417200

B 2.47971200 -1.31543300 1.54417200

B 3.23981700 -0.88171100 -1.26618700

B -3.23981700 0.88171100 -1.26618700

B -0.88420100 -2.24223000 1.80733500

B 3.23981700 0.88171100 -1.26618700

B 2.71582200 0.00000000 2.46874400

B -1.83694800 2.55615200 0.54604400

B -0.88420100 2.24223000 1.80733500

B 0.88420100 2.24223000 1.80733500

B 1.91262300 -2.12790600 -1.03586900

B -1.91262300 2.12790600 -1.03586900

B 1.91262300 2.12790600 -1.03586900

B 1.42522400 -0.91360000 2.84069600

B 1.42522400 0.91360000 2.84069600

B 3.15106100 1.75332400 0.06328500

B 1.94304600 1.25316300 -2.52575500

B -0.81258400 -2.30918600 -2.24474700

B -3.23981700 -0.88171100 -1.26618700

B -1.42522400 -0.91360000 2.84069600

B -1.42522400 0.91360000 2.84069600

B -1.94304600 1.25316300 -2.52575500

B 1.83694800 -2.55615200 0.54604400

B -2.71582200 0.00000000 2.46874400

B 1.94304600 -1.25316300 -2.52575500

B -3.00151700 0.00000000 -2.59934800

B -1.83694800 -2.55615200 0.54604400

B -1.94304600 -1.25316300 -2.52575500

B 0.00000000 0.85926300 -2.54367600

B 0.81258400 2.30918600 -2.24474700

B 0.00000000 -0.85926300 -2.54367600

B 0.81258400 -2.30918600 -2.24474700

Na 0.00000000 0.00000000 0.14321000

C_s_ K&B_44_^−^ (**I**)

B 0.29788000 -2.12145700 3.14999900

B -2.64813000 -2.59506800 0.00000000

B -1.22099200 -1.93389800 2.46239300

B -0.35081000 1.36034700 -3.16990100

B 2.57402000 0.11457300 -2.98468100

B 2.67912500 -2.24133100 0.81229400

B -2.15388600 1.53320700 -0.89043700

B 0.63066200 1.92298100 1.88920000

B -0.35081000 1.36034700 3.16990100

B -3.27727500 0.75481000 0.00000000

B -1.71218400 0.64352000 2.46208400

B -0.03529400 -3.01365100 -1.85553800

B -1.22099200 -1.93389800 -2.46239300

B -1.71218400 0.64352000 -2.46208400

B 1.11081900 0.73796100 -3.23979600

B 1.42409200 -0.99839500 3.20930800

B -2.15388600 1.53320700 0.89043700

B 1.42409200 -0.99839500 -3.20930800

B -2.37257500 -0.81019500 -2.69528700

B -0.03529400 -3.01365100 1.85553800

B -1.31682800 -2.91460000 0.88500200

B -1.31682800 -2.91460000 -0.88500200

B 0.63066200 1.92298100 -1.88920000

B 1.42954100 -2.26950700 1.89325000

B 1.42954100 -2.26950700 -1.89325000

B -2.90821800 0.01609200 -1.40749800

B -2.55528700 -1.76791000 -1.39447700

B 0.29788000 -2.12145700 -3.14999900

B 2.73305100 -1.13456800 -1.92308600

B 1.81843600 2.34232000 0.82175300

B 1.11081900 0.73796100 3.23979600

B -2.90821800 0.01609200 1.40749800

B -2.55528700 -1.76791000 1.39447700

B 2.73305100 -1.13456800 1.92308600

B -1.01127800 2.08030800 -1.89988300

B -2.37257500 -0.81019500 2.69528700

B 2.26791300 1.34024400 -1.94488700

B 2.57402000 0.11457300 2.98468100

B -1.01127800 2.08030800 1.89988300

B 2.26791300 1.34024400 1.94488700

B 2.55982800 -0.76087900 0.00000000

B 2.67912500 -2.24133100 -0.81229400

B 2.23835600 0.92755100 0.00000000

B 1.81843600 2.34232000 -0.82175300

K -0.40241000 4.18460600 0.00000000

C_2v_ K@B_44_^−^ (**I**)

B -3.14777800 1.77738200 0.06293000

B 0.00000000 1.69587000 3.05551600

B -2.47218900 1.34599900 1.54019900

B 3.14777800 -1.77738200 0.06293000

B 2.99004700 0.00000000 -2.58317000

B -0.81106600 2.31684600 -2.25286100

B 0.89178200 -2.28731400 1.81980900

B -1.91182100 -2.16214100 -1.03920000

B -3.14777800 -1.77738200 0.06293000

B 0.00000000 -1.69587000 3.05551600

B -2.47218900 -1.34599900 1.54019900

B 1.84640900 2.59803400 0.55044200

B 2.47218900 1.34599900 1.54019900

B 2.47218900 -1.34599900 1.54019900

B 3.22728900 -0.88172800 -1.25024100

B -3.22728900 0.88172800 -1.25024100

B -0.89178200 -2.28731400 1.81980900

B 3.22728900 0.88172800 -1.25024100

B 2.69220300 0.00000000 2.42331700

B -1.84640900 2.59803400 0.55044200

B -0.89178200 2.28731400 1.81980900

B 0.89178200 2.28731400 1.81980900

B 1.91182100 -2.16214100 -1.03920000

B -1.91182100 2.16214100 -1.03920000

B 1.91182100 2.16214100 -1.03920000

B 1.41392700 -0.91958800 2.82435100

B 1.41392700 0.91958800 2.82435100

B 3.14777800 1.77738200 0.06293000

B 1.94018700 1.25965600 -2.51870200

B -0.81106600 -2.31684600 -2.25286100

B -3.22728900 -0.88172800 -1.25024100

B -1.41392700 -0.91958800 2.82435100

B -1.41392700 0.91958800 2.82435100

B -1.94018700 1.25965600 -2.51870200

B 1.84640900 -2.59803400 0.55044200

B -2.69220300 0.00000000 2.42331700

B 1.94018700 -1.25965600 -2.51870200

B -2.99004700 0.00000000 -2.58317000

B -1.84640900 -2.59803400 0.55044200

B -1.94018700 -1.25965600 -2.51870200

B 0.00000000 0.87002800 -2.60165300

B 0.81106600 2.31684600 -2.25286100

B 0.00000000 -0.87002800 -2.60165300

B 0.81106600 -2.31684600 -2.25286100

K 0.00000000 0.00000000 0.12238700

C_1_ Li&B_44_^−^ (**IV**)

B 1.22396300 0.64884000 -3.15036200

B -0.34637800 0.74617400 -3.73551100

B -1.53985600 2.31894200 -1.50778700

B -0.17130200 2.98241500 -1.04552300

B 2.78677200 1.45274400 -0.92915600

B 1.97574100 1.94053800 0.56490000

B -1.12122200 2.47493300 0.27218000

B -1.21994600 -0.59196900 -3.15582600

B 1.58226700 -1.12786300 -2.83468900

B 1.36928500 2.41624200 -1.11755200

B 0.54982800 2.67604000 0.35643000

B -1.60842000 1.20001700 -2.84487000

B 0.34065100 -0.67558300 -3.74767000

B 2.13145700 1.74495500 -2.39780000

B 3.34936000 1.05416700 0.50858300

B 2.59165400 0.20029300 -2.21389200

B -2.62918900 1.73322300 -0.21286200

B -2.84660000 1.42352700 -1.82194700

B -2.58566000 -0.14737800 -2.21225500

B 1.06500200 -2.44346500 0.25957500

B -0.58196900 -2.73527700 0.28896600

B -1.82439500 -0.58139500 3.37091400

B 1.18056900 -0.58132300 3.89072400

B 2.09123700 -1.82800500 1.36588500

B 1.92076500 0.78046500 3.37124500

B -2.18813400 0.79898800 2.49770100

B -1.35842300 -2.40166200 -1.17066400

B 1.54739500 -2.31454500 -1.53789800

B 2.15071900 -0.66864500 2.52625100

B -1.10265900 0.84197700 3.77574200

B -1.97196600 -1.93417200 0.55781300

B 0.18002400 -3.00177200 -1.09775200

B 2.94517500 -0.46529900 1.02016600

B 2.54104100 -1.62603200 -0.19607300

B -2.96658500 0.53624400 0.99972900

B -3.29500300 -0.99869700 0.51693500

B -2.53181200 -0.86064800 1.97067800

B -2.74225700 -1.39254400 -0.93082300

B -2.23570600 1.96136000 1.35126500

B 2.66625800 0.97211900 1.98176600

B 2.81699300 -1.36936800 -1.80472100

B -2.12786000 -1.69513500 -2.41411500

B -0.37448900 -0.57643900 4.20977400

B 0.43053300 0.86736400 4.13265400

Li -0.06685400 -1.75435300 2.28987600

C_1_ Na&B_44_^−^ (**IV**)

B 3.28356800 1.25669300 -0.37735000

B 3.88771100 -0.30921200 -0.40648500

B 1.85679300 -1.52486100 -2.20957100

B 1.45794900 -0.16126900 -2.92310300

B 1.15193700 2.79777700 -1.42128600

B -0.27411600 1.97026000 -2.05354700

B 0.10390600 -1.12532000 -2.55363100

B 3.17327100 -1.18574300 0.85933500

B 2.76461800 1.61203500 1.35360200

B 1.45762700 1.37968900 -2.35407200

B 0.02364400 0.54368000 -2.75821700

B 3.06478700 -1.58088500 -0.95333400

B 3.73421100 0.37898800 1.00739300

B 2.65001700 2.15846900 -1.55124400

B -0.32191600 3.34752200 -1.17282800

B 2.29716300 2.62014100 -0.03749700

B 0.51622000 -2.63186600 -1.76833600

B 2.08305800 -2.82932900 -1.28235800

B 2.29119700 -2.55973700 0.32109500

B -0.44287100 1.07185900 2.32155000

B -0.48111200 -0.57907700 2.63322800

B -3.31739500 -1.84887600 0.11475500

B -3.86530100 1.15246100 0.03994000

B -1.49590500 2.08889200 1.59642800

B -3.18337900 1.90269600 -1.23938100

B -2.29135200 -2.23456500 -1.13837500

B 1.00858900 -1.34017900 2.45520000

B 1.34640200 1.57831600 2.39583600

B -2.51868300 2.12503900 0.30666100

B -3.52495100 -1.13108600 -1.35185600

B -0.65402300 -1.96781000 1.80997000

B 0.85768000 0.20471900 3.03414000

B -1.01291000 2.95213800 0.28044700

B 0.07938800 2.54998300 1.55147300

B -0.82159200 -2.97198700 -0.70160900

B -0.51356600 -3.28398600 0.88026600

B -1.95698600 -2.55454300 0.56935000

B 0.88289200 -2.72343000 1.42272200

B -1.01495500 -2.25055700 -2.16132100

B -1.79278200 2.66118800 -1.25879100

B 1.70608800 2.84146200 1.47391900

B 2.32377100 -2.09526700 1.87924000

B -4.17833500 -0.41076300 -0.01348600

B -3.88612200 0.39829800 -1.42307800

Na -2.93374300 -0.13270800 2.18373000

C_2_ Na@B_44_^−^ (**IV**)

B 1.06780700 0.86522600 -3.16106500

B -0.48451500 0.62255600 -3.74103600

B -1.99003500 1.92420800 -1.51723500

B -0.78808100 2.86239200 -1.05830000

B 2.42431800 1.97354500 -0.94691700

B 1.55809600 2.30105000 0.55056600

B -1.59966400 2.15889800 0.26894200

B -1.06780700 -0.86522600 -3.16106500

B 1.80811600 -0.80622800 -2.84601800

B 0.83874100 2.62942700 -1.15129800

B 0.00000000 2.68892700 0.33661400

B -1.80811600 0.80622800 -2.84601800

B 0.48451500 -0.62255600 -3.74103600

B 1.73333100 2.12916900 -2.42117800

B 3.06918100 1.70152800 0.48877900

B 2.50815900 0.71529400 -2.23521100

B -2.90103400 1.08712300 -0.21397100

B -3.07158400 0.77017400 -1.82708000

B -2.50815900 -0.71529400 -2.23521100

B 1.59966400 -2.15889800 0.26894200

B 0.00000000 -2.68892700 0.33661400

B -1.71990900 -1.06396500 3.34857100

B 1.27647700 -0.46295900 3.77716500

B 2.54743800 -1.39376900 1.35708500

B 1.71990900 1.06396500 3.34857100

B -2.38128300 0.25586000 2.52619900

B -0.83874100 -2.62942700 -1.15129800

B 1.99003500 -1.92420800 -1.51723500

B 2.38128300 -0.25586000 2.52619900

B -1.27647700 0.46295900 3.77716500

B -1.55809600 -2.30105000 0.55056600

B 0.78808100 -2.86239200 -1.05830000

B 3.06844100 0.13332800 1.01225400

B 2.90103400 -1.08712300 -0.21397100

B -3.06844100 -0.13332800 1.01225400

B -3.06918100 -1.70152800 0.48877900

B -2.43730900 -1.44415900 1.97854000

B -2.42431800 -1.97354500 -0.94691700

B -2.54743800 1.39376900 1.35708500

B 2.43730900 1.44415900 1.97854000

B 3.07158400 -0.77017400 -1.82708000

B -1.73333100 -2.12916900 -2.42117800

B -0.23818800 -0.79898900 4.06653800

B 0.23818800 0.79898900 4.06653800

Na 0.00000000 0.00000000 1.28005100

C_1_ K&B_44_^−^ (**IV**)

B 3.45458900 1.22130300 0.08538700

B 4.03869400 -0.35033700 0.12143700

B 2.31006600 -1.50158700 -2.00471500

B 2.04349000 -0.11950400 -2.74440500

B 1.53838300 2.81672100 -1.25212700

B 0.22100800 2.02099300 -2.11540700

B 0.63872200 -1.07378000 -2.61274300

B 3.12572200 -1.24198600 1.24074100

B 2.67037900 1.55363900 1.72055500

B 1.97036400 1.41235500 -2.15530300

B 0.60656100 0.59966600 -2.78868700

B 3.30263100 -1.60083000 -0.57619800

B 3.66973200 0.31353300 1.50734600

B 3.03010200 2.15608800 -1.15453600

B 0.04937400 3.38136500 -1.22309600

B 2.44559700 2.59450300 0.29267700

B 0.90687600 -2.60332800 -1.80886900

B 2.37369400 -2.82927800 -1.08357400

B 2.32584800 -2.59371600 0.53810200

B -0.65017100 1.03935500 2.14474700

B -0.75236900 -0.61559700 2.39996400

B -3.16536100 -1.78580500 -0.54824400

B -3.66972900 1.21584400 -0.66119600

B -1.57354100 2.07616800 1.28700500

B -2.77701500 1.98409600 -1.79212300

B -1.96370300 -2.18789800 -1.62703000

B 0.74176000 -1.39124000 2.46308200

B 1.09946500 1.52326900 2.51897000

B -2.37497300 2.15935000 -0.14931400

B -3.10913200 -1.04528600 -2.01965400

B -0.81477900 -1.98698400 1.54454100

B 0.50531000 0.14164700 3.04248100

B -0.87795800 2.96896500 0.08829100

B -0.00183100 2.52518900 1.49917000

B -0.59080100 -2.94905800 -0.97724600

B -0.54020300 -3.28893100 0.62680700

B -1.90895900 -2.53094600 0.10559600

B 0.75714900 -2.75632300 1.39755800

B -0.53722500 -2.19422500 -2.43095300

B -1.39590100 2.72409100 -1.56479300

B 1.62119000 2.79635400 1.69245700

B 2.11704900 -2.16363200 2.09247800

B -3.98019800 -0.34144000 -0.80012500

B -3.43366500 0.48900000 -2.12075300

K -3.53848500 -0.14783700 2.05307800

C_2_ K@B_44_^−^ (**IV**)

B 1.05843000 0.85660600 -3.12883700

B -0.48421500 0.62462500 -3.72149800

B -1.97057500 1.98789000 -1.47840600

B -0.79872000 2.97979700 -1.03730500

B 2.35916100 1.97101800 -0.88560300

B 1.54328800 2.41205200 0.60486300

B -1.60978800 2.31354200 0.30809800

B -1.05843000 -0.85660600 -3.12883700

B 1.78215100 -0.82906300 -2.79143600

B 0.83250900 2.74333000 -1.12764700

B 0.00000000 2.88432500 0.36112200

B -1.78215100 0.82906300 -2.79143600

B 0.48421500 -0.62462500 -3.72149800

B 1.69163300 2.12472900 -2.37154600

B 2.97740300 1.65194500 0.54369300

B 2.46425700 0.70789200 -2.15765700

B -2.85927500 1.15677400 -0.14911300

B -3.01976800 0.78508800 -1.74749000

B -2.46425700 -0.70789200 -2.15765700

B 1.60978800 -2.31354200 0.30809800

B 0.00000000 -2.88432500 0.36112200

B -1.66771800 -1.04358000 3.41680100

B 1.26073100 -0.47471700 3.89955800

B 2.46682800 -1.45082700 1.40734300

B 1.66771800 1.04358000 3.41680100

B -2.26060900 0.29540100 2.56072200

B -0.83250900 -2.74333000 -1.12764700

B 1.97057500 -1.98789000 -1.47840600

B 2.26060900 -0.29540100 2.56072200

B -1.26073100 0.47471700 3.89955800

B -1.54328800 -2.41205200 0.60486300

B 0.79872000 -2.97979700 -1.03730500

B 2.99554100 0.08427300 1.07494900

B 2.85927500 -1.15677400 -0.14911300

B -2.99554100 -0.08427300 1.07494900

B -2.97740300 -1.65194500 0.54369300

B -2.33897300 -1.39182100 2.02462200

B -2.35916100 -1.97101800 -0.88560300

B -2.46682800 1.45082700 1.40734300

B 2.33897300 1.39182100 2.02462200

B 3.01976800 -0.78508800 -1.74749000

B -1.69163300 -2.12472900 -2.37154600

B -0.24439000 -0.79545800 4.22044400

B 0.24439000 0.79545800 4.22044400

K 0.00000000 0.00000000 0.09174900
